# In-depth analysis of isochorismate synthase-derived metabolism in plant immunity: Identification of *meta*-substituted benzoates and salicyloyl-malate

**DOI:** 10.1016/j.jbc.2024.107667

**Published:** 2024-08-12

**Authors:** Nicola Scholten, Michael Hartmann, Sarah Abts, Laura Abts, Elke Reinartz, Angelo Altavilla, Thomas J.J. Müller, Jürgen Zeier

**Affiliations:** 1 Department of Biology, Institute for Molecular Ecophysiology of Plants, Heinrich Heine University, Düsseldorf, Germany; 2 Department of Chemistry, Institute of Organic Chemistry and Macromolecular Chemistry, Heinrich Heine University, Düsseldorf, Germany; 3 Cluster of Excellence on Plant Sciences (CEPLAS), Heinrich Heine University, Düsseldorf, Germany

**Keywords:** salicylic acid derivatives, isochorismate synthase, ICS1, EDS5, PBS3, NPR1, salicyloyl-malate, *meta*-substituted aromatics, *Arabidopsis thaliana*, plant immunity

## Abstract

Isochorismate-derived metabolism enables biosynthesis of the plant defense hormone salicylic acid (SA) and its derivatives. In *Arabidopsis thaliana*, the stress-induced accumulation of SA depends on ISOCHORISMATE SYNTHASE1 (ICS1) and also requires the presumed isochorismate transporter ENHANCED DISEASE SUSCEPTIBILITY5 (EDS5) and the GH3 enzyme avrPphB SUSCEPTIBLE3 (PBS3). By comparative metabolite and structural analyses, we identified several hitherto unreported *ICS1-* and *EDS5*-dependent, biotic stress-inducible Arabidopsis metabolites. These involve *meta*-substituted SA derivatives (5-formyl-SA, 5-carboxy-SA, 5-carboxymethyl-SA), their benzoic acid (BA) analogs (3-formyl-BA, 3-carboxy-BA, 3-carboxymethyl-BA), and besides the previously detected salicyloyl-aspartate (SA-Asp), the ester conjugate salicyloyl-malate (SA-Mal). SA functions as a biosynthetic precursor for SA-Mal and SA-Asp, but not for the *meta*-substituted SA- and BA-derivatives, which accumulate to moderate levels at later stages of bacterial infection. Interestingly, Arabidopsis leaves possess oxidizing activity to effectively convert *meta*-formyl- into *meta*-carboxy-SA/BAs. In contrast to SA, exogenously applied *meta*-substituted SA/BA-derivatives and SA-Mal exert a moderate impact on plant immunity and defence-related gene expression. While the isochorismate-derived metabolites are negatively regulated by the SA receptor NON-EXPRESSOR OF PR GENES1, SA conjugates (SA-Mal, SA-Asp, SA-glucose conjugates) and *meta*-substituted SA/BA-derivatives are oppositely affected by PBS3. Notably, our data indicate a PBS3-independent path to isochorismate-derived SA at later stages of bacterial infection, which does not considerably impact immune-related characteristics. Moreover, our results argue against a previously proposed role of EDS5 in the biosynthesis of the immune signal N-hydroxypipecolic acid and associated transport processes. We propose a significantly extended biochemical scheme of plant isochorismate metabolism that involves an alternative generation mode for benzoate- and salicylate-derivatives.

Plants and many microorganisms can synthesize the aromatic amino acids phenylalanine, tyrosine, and tryptophan from erythrose-4-phosphate and phosphoenolpyruvate *via* the shikimate pathway (1). Chorismate is a key branch-point intermediate of this metabolic route. It can be enzymatically converted to prephenate by chorismate mutase toward the production of Phe and Tyr, to anthranilate for Trp biosynthesis by anthranilate synthase, and to aminodeoxychorismate by aminodeoxychorismate synthase, a precursor of the cofactor tetrahydrofolate (2, 3). In addition, isochorismate synthase (ICS) catalyzes the rearrangement of chorismate to isochorismate, a central precursor for the biosynthesis of salicylic acid (SA) and the photosystem I-resident redox cofactor phylloquinone (4, 5, 6, 7).

SA is a central plant stress hormone that mediates inducible defense responses associated with pattern- and effector-triggered immunity (8). Moreover, in an interplay with the L-Lys-derived immune signal *N*-hydroxypipecolic acid (NHP), SA guarantees the establishment of systemic acquired resistance (SAR), a state of primed immunity that develops throughout the plant foliage in response to a localized leaf inoculation (9, 10, 11, 12). SA-mediated defense responses require the transcriptional co-activator NON-EXPRESSOR OF PR GENES1 (NPR1) (8). The recognition of pathogens triggers SA biosynthesis (13), and the consequentially increased levels of SA result in the translocation of NPR1 from the cytosol to the nucleus (14). The binding of SA to NPR1 then induces the expression of a battery of defense-related genes for immune activation (15, 16).

Basically, plants are able to synthesize SA by two metabolic routes—the ICS and the phenylalanine ammonia lyase (PAL) pathways (17). The accumulation of SA in pathogen-inoculated Arabidopsis leaves essentially proceeds *via* the ICS pathway, which is initiated by the conversion of chorismate to isochorismate in the chloroplast *via* the stress-inducible ICS isoform ISOCHORISMATE SYNTHASE1 (ICS1) (5, 6, 18, 19). Although bacteria directly convert isochorismate to SA *via* isochorismate pyruvate lyase (IPL) activities (20), plants pursue a circuit strategy for this conversion (21, 22). The Arabidopsis Gretchen Hagen 3 (GH3) acyl acid amido synthetase avrPphB SUSCEPTIBLE3 (PBS3) first catalyzes the ATP-consuming conjugation of isochorismate and *L*-Glu to generate isochorismoyl-*L*-glutamate (IC-Glu) as an intermediate. Elimination of N-pyruvoyl-*L*-glutamate (Pyr-Glu) from IC-Glu, either spontaneously or by the action of the acyltransferase ENHANCED *PSEUDOMONAS* SUSCEPTIBILTY1, then results in SA formation. Since PBS3 is located in the cytosol, transport of isochorismate out of the chloroplast is necessary for the PBS3-mediated biosynthesis of SA. The chloroplast envelope-resident multidrug and toxin extrusion (MATE) transporter ENHANCED DISEASE SUSCEPTIBILITY5 (EDS5) has been implicated in this transport process (21). Just as *ICS1*, *EDS5* is required for the biotic stress-induced biosynthesis of SA in Arabidopsis (18, 23). Initially, assays with isolated chloroplasts suggested that EDS5 might enable the transport of SA out of the chloroplast (24). However, more recent results on the metabolic phenotypes of Arabidopsis lines expressing differentially targeted ICS1- and PBS3-fusion proteins in the *eds5* mutant background strongly suggest a function for EDS5 in isochorismate transport out of the chloroplast (21). Therefore, the current model of SA biosynthesis *via* the ICS pathway involves the ICS1-catalyzed isomerization of chorismate to isochorismate in the chloroplast, EDS5-mediated transport of isochorismate across the chloroplast envelope, and PBS3-catalysed formation of IC-Glu, which subsequently decomposes to Pyr-Glu and SA (9).

The biochemical modification of a plant hormone generally alters its physiological activity. The glucosylation of SA to the biologically inactive SA-β-glucoside (SAG) and to SA glucose ester (SGE) are predominant SA metabolic pathways. Remarkably, the uridine diphosphate-dependent glycosyltransferase (UGT) UGT76B1 is responsible for the concerted glucosylation of both SA to SAG and NHP to NHP glucoside (NHPG) under biotic stress conditions, respectively, and in this way terminates SAR signaling (9, 25, 26). Two other UGTs, UGT74F1 and UGT74F2, contribute to basal SAG levels, and UGT74F2 also exhibits SGE-forming activity (27). Another prominent option of SA modification is its hydroxylation to 2,3-dihydroxybenzoic acid (2,3-DHBA) and 2,5-DHBA (gentisic acid), which is mediated by the 2-oxoglutarate-dependent dioxygenases SA-3-hydroxylase (S3H) and SA-5-hydroxylase (S5H), respectively (28, 29). The final metabolic products of the SA hydroxylation pathway are DHBA glucosides and xylosides (30, 31, 32). Moreover, SA is converted to its volatile methyl ester methyl salicylate (MeSA) by the jasmonic acid-inducible methyltransferase BENZOIC ACID SALICYLIC ACID METHYLTRANSFERASE1 (BSMT1) (33, 34). MeSA is a significant constituent of the floral scents of many plant species (35). Although MeSA has been suggested as a mobile signal for SAR in tobacco (36), the SAR-inducing abilities of MeSA-deficient Arabidopsis *bsmt1* mutant plants argue against a general function for MeSA in SAR establishment (34). Finally, the aspartyl amide conjugate of SA, salicyloyl-aspartate (SA-Asp), was detected as a natural SA derivative in different plant species (37, 38, 39). *In vitro* biochemical assays suggest a role for the acyl acid amido synthetase GH3.5 in the formation of SA-Asp (40, 41, 42).

In the present study, we identify the occurrence of several hitherto undescribed SA and benzoic acid (BA) derivatives in leaf extracts of Arabidopsis which accumulate upon bacterial inoculation in an *ICS1*- and *EDS5*-dependent manner. These include two related sets of *meta-*substituted SA and BA derivatives carrying formyl-, carboxy- and carboxymethyl-moieties in their aromatic ring. Moreover, we identify the malate ester of SA, salicyloyl-malate (SA-Mal), as a natural SA derivative that accumulates together with SA-Asp and the SA biosynthesis-related metabolites IC-Glu and Pyr-Glu in pathogen-inoculated leaves in dependency of *PBS3*. We provide information about the biosynthetic origins of these substances, characterize their time-dependent accumulation following bacterial inoculation, and study their immune-inducing abilities. Our data further indicate the existence of an ICS1-dependent but PBS3-independent path to isochorismate-derived SA in Arabidopsis that operates at later stages of bacterial infection to some extent. We further show that *ics1*, *eds5*, and *pbs3* knockout mutants show similar immune-related characteristics, suggesting that early production of ICS pathway-derived SA functions as a major determinant of plant immune responses. Our results also exclude a previously suggested function for EDS5 in NHP biosynthesis and putatively associated transport processes. By integrating our findings, we propose an extended biochemical scheme of isochorismate synthase-derived metabolism in plants.

## Results

### Identification of novel *meta*-substituted salicylic acid and benzoic acid derivatives in *Arabidopsis thaliana* leaves that accumulate upon pathogen inoculation in dependency of isochorismate synthase

**Figure 1:**
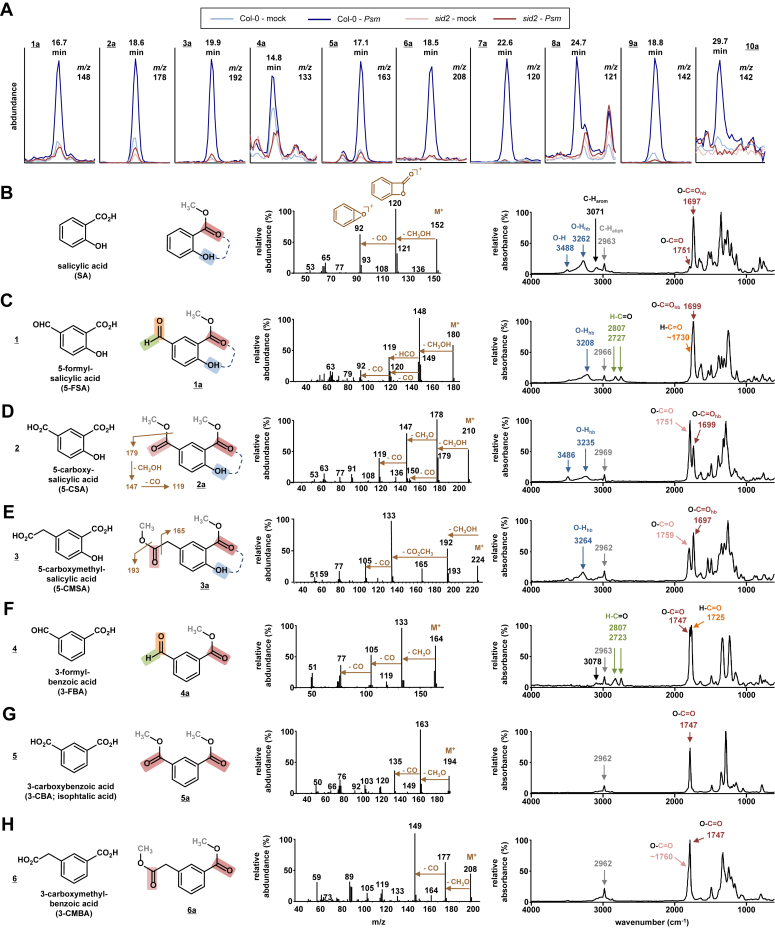
**Identification of *meta*-substituted salicylic acid (SA) and benzoic acid (BA) derivatives that accumulate in Arabidopsis leaves in dependence of *ICS1*.***A*, segments of overlaid ion chromatograms of extract samples from *Arabidopsis thaliana* Col-0 wildtype (*blue*) and *sid2* mutant (*red*) leaves, as analysed by GC-MS and GC-FTIR. The *sid2* mutant is defective in the *ISOCHORISMATE SYNTHASE1* (*ICS1*) gene. Leaf samples were harvested 48 h after inoculation with *Pseudomonas syringae* pv. *maculicola* (*Psm*; *dark colors*) or 48 h after a mock-control infiltration (mock; *light colors*). Samples were derivatised with trimethylsilyl-diazomethane to convert carboxylic acid groups occurring in analytes into methyl ester groups prior to GC-based analysis. Retention times and *m/z* values of ion chromatograms for analytes (1a to 10a) accumulating in Col-0 but not in *sid2* upon pathogen inoculation are given. *B*–*H*, identification of six *meta*-substituted SA and BA derivatives. Columns from *left* to *right*: First column: names, structural formulas, and assigned numerical identifiers (1–6) of analytes with compromised accumulation in *sid2*. Second column: structural formulas of their derivatised forms (1a to 6a), as detected by GC-MS and GC-FTIR. Methyl groups depicted in *grey* are introduced by sample derivatisation. Dashed lines in formulas indicate the occurrence of hydrogen bonds in SA derivatives. Third column: Mass spectra of the substances recorded in the GC-MS analysis. Fragmentation patterns and proposed structures of ions are indicated in brown (M^+^: molecular ion). Forth column: Infrared (IR) spectra of the substances analysed by GC-FTIR. The wavenumbers ṽ (in cm^−1^) of key IR vibrations and their assignments to functional groups are highlighted. The color assignments correspond to the color shadings of specific functional groups depicted in the molecular structures (second column) to highlight their IR vibrational characteristics [O-H: O-H stretching vibration (*blue*); C=O: C=O stretching vibration of aromatic methyl ester (O-C=O) (*dark red*), aliphatic methyl ester (*light red*), or aromatic aldehyde (H-C=O) (*orange*); C-H: aromatic C-H-stretching (*black*), aliphatic C-H-stretching (*grey*), or C-H stretching of aldehyde group (H-C=O, *green*), The subscript “hb” indicates the involvement of OH- or C=O-groups in hydrogen bonds]. *B*, salicylic acid (SA). *C*, 5-formyl-salicylic acid (5-FSA, 1). *D*, 5-carboxy-salicylic acid (5-CSA, 2). *E*, 5-carboxymethyl-salicylic acid (5-CMSA, 3). *F*, 3-formyl-benzoic acid (3-FBA, 4). *G*, 3-carboxy-benzoic acid (3-CBA, 5). *H*, 3-carboxymethyl-benzoic acid (3-CMBA, 6). The identities of analytes (1, 2, 4, 5, and 6) were further confirmed by use of commercially available authentic substances (Fig. S1). Moreover, 1 to 6 were identified in plant extracts using LC-qTOF/MS-based analysis (Fig. S2).

The plastid-resident isochorismate synthase catalyzes the conversion of chorismate to isochorismate in plants (5, 6). The *ICS1* gene, which is defective in the SA-biosynthetic mutant *salicylic acid-induction-deficient2* (*sid2*) (18), is the biotic stress-inducible isochorismate synthase isoform of Arabidopsis (4, 19). To get further insights into *ICS1*-dependent metabolism, we inoculated leaves of wild-type Arabidopsis Col-0 and *sid2* mutant plants with the compatible bacterial pathogen *Pseudomonas syringae* pv. *maculicola* ES 4326 (*Psm*), harvested leaves at 48 h post-treatment (hpt), and performed comparative gas chromatography-mass spectrometry (GC-MS)-based metabolite analyses of leaf extracts (Fig. 1). We thereby applied an analytical method that involves sample derivatization with trimethylsilyl-diazomethane to convert analytes with free carboxylic acid groups into methyl esters and thereby facilitate GC-based analyses (43, 44). Analyses of GC-MS ion chromatograms of distinct mass-to-charge ratios (*m/z*) identified, in addition to the previously analyzed SA and SA glucose conjugates (11), ten peaks of analytes (1a to 10a) that accumulated in the Col-0 wild-type but not in *sid2* upon *Psm*-inoculation (Fig. 1*A*).

**Figure 2:**
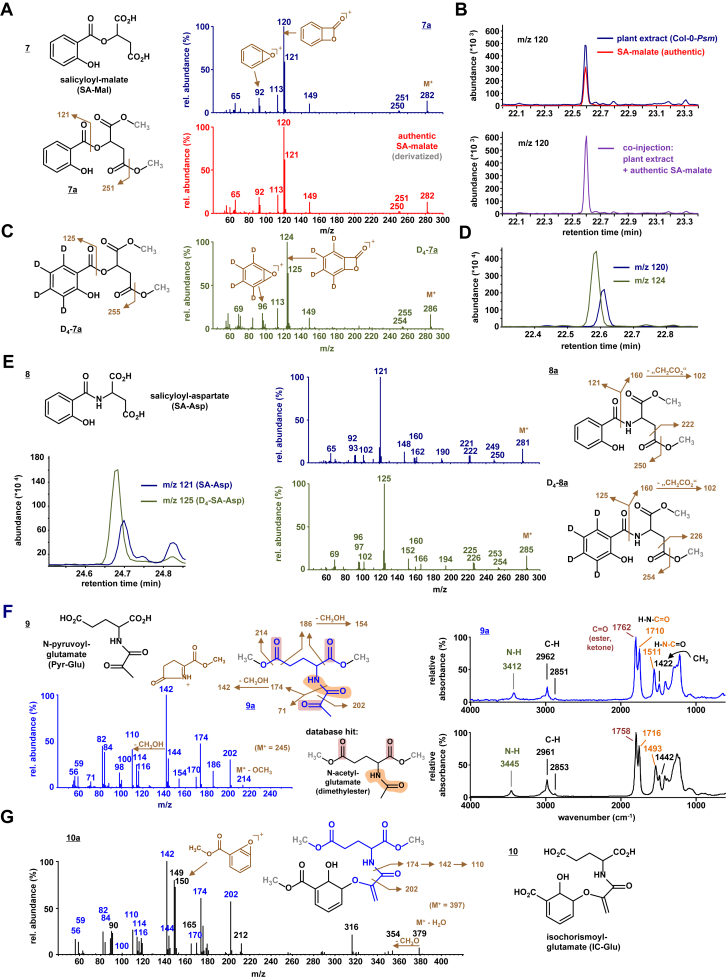
**Identification of *ICS1*- and *PBS3*-dependent SA-malate, SA-aspartate, and SA biosynthesis-related compounds.***A*–*G*, chemical characterization of plant-derived compounds 7 to 10 by GC-MS- and/or GC-FTIR-based analyses. *A*–*D*, identification of salicyloyl-malate (SA-Mal, 7) as a natural product of Arabidopsis accumulating in *Psm*-inoculated leaves by GC-MS. *A*, mass spectra of the plant-derived compound 7a (*blue*) and of derivatized, synthetic SA-Mal (*red*) are identical. Fragmentation patterns of derivatized SA-Mal and proposed fragment structures are indicated in *brown* (M^+^: molecular ion). Methyl groups appearing in the structural formula in *grey* are introduced by derivatization. *B*, retention times of plant-derived, derivatized 7a, and synthetic SA-Mal are identical. *Top*: Overlaid ion chromatograms (*m/z* = 120) of GC-MS-analysed samples of extracts from *Psm*-inoculated Col-0 leaves (*blue*) and of authentic, derivatized SA-Mal (*red*). *Bottom*: Co-injection of plant extract sample and authentic SA-Mal results in a single peak in the ion chromatogram (*purple*). *C* and *D*, *Psm*-inoculated Arabidopsis leaves synthesize D_4_-labeled SA-Mal from exogenously supplemented deuterated SA (Fig. S4). *C*, the mass spectrum of derivatized D_4_-SA-Mal (D_4_-7a) generated in leaves after co-infiltration of *Psm* and 0.5 mM D_4_-SA shows a characteristic shift of 4 mass units compared with the mass spectrum of unlabeled SA-Mal (*A*). Proposed fragmentations are indicated in *brown*. *D*, ion chromatograms indicating the presence of both unlabeled SA-Mal (7a) (*green*) and D_4_-SA-Mal (*blue*) in extracts of leaves co-infiltrated with 0.5 mM D_4_-SA and *Psm*. *E*, GC-MS-based analysis indicates that compound 8a represents salicyloyl-aspartate (SA-Asp, 8). The mass spectrum of compound 8a (*blue*) displays an M^+^ ion at *m/z* 281, as expected for derivatised (methylated) SA-Asp. Further, the occurrence of α-cleavage-derived main fragments of *m/z* 121, 160, 222, and 250 confirm the structure of 8a as derivatized SA-Asp. Co-infiltration of D_4_-SA and *Psm* into leaves generates D_4_-labeled SA-Asp (*green* ion chromatogram and mass spectrum) in addition to unlabeled SA-Asp (*blue*), indicating that, similar to SA-Mal (*C* and *D*), SA-Asp is directly synthesized from SA in plants. The fragmentation pattern of the deuterium-labeled compound (D_4_-8a) is again consistent with the assigned D_4_-SA-Asp structure. SA-Mal (7) and SA-Asp (8) were also identified and analyzed in plant extracts *via* LC-qTOF/MS analysis (Fig. S2). *F* and *G*, spectroscopic characterization strongly suggests that the analytes 9a and 10a (Fig. 1*A*) correspond to derivatized N-pyruvoyl-glutamate and isochorismoyl-glutamate, respectively. *F*, based on mass spectrometric and IR spectroscopic characterization, analyte 9a was identified as derivatized (dimethylated) N-pyruvoyl-glutamate (Pyr-Glu, 9). *Left*: The mass spectrum of 9a contains a prominent m/z *174* ion that can result from cleavage of the amide-bond of Pyr-Glu, which decomposes to the main fragment at *m/z* 142 by loss of CH_3_OH and possible cyclization into the indicated pyrrolinone. Other plausible fragmentations, including the loss of acetyl- and methoxyl-moieties to yield *m/z* 202 (M^+^-43) and 214 (M^+^-31), respectively, are indicated. *Right*: The IR spectrum of the proposed dimethylated Pyr-Glu (9a) (*blue*) confirms the presence of the amide bond [ṽ ∼1710 (C=O), ṽ ∼1500 (C-N) and ṽ ∼3400 N-H; *orange*], carbonyl groups within methylester and keto moieties [ṽ ∼1760 (C=O); *red*], and aliphatic methyl (ene) units [ṽ ∼2960 and 2850 (C-H)] in the molecule, and the high similarity of the recorded IR spectrum with the best database hit, the IR spectrum of N-acetyl-glutamate dimethylester (*black*), further substantiated the notion that 9a represents dimethylated Pyr-Glu. *G*, the mass spectrum of analyte 10a contained the fragment series *m/z* 202, 174, and 142 observed for Pyr-Glu, and a plausible M^+^-H_2_O ion is consistent with a putative derivatized (trimethylated) isochorismoyl-9-glutamate (IC-Glu).

To elucidate the chemical structures of these analytes, we recorded their mass spectra and interpreted molecular ions (M^+^) and mass spectral fragmentation patterns. In addition, we analyzed the derivatized plant extract samples by GC-Fourier transform infrared spectroscopy (FTIR) and managed to obtain infrared (IR) spectra for most of the analytes (Figs. 1 and 2). The mass spectrum of the well-known *ICS1*-dependent metabolite SA (which is derivatized to SA methyl ester by the applied method) shows an M^+^ of 152 and characteristic fragment ions of *m/z* 120 and *m/z* 92, carrying predicted lactone- and epoxy-groups at the aromatic ring whose formation is due to subsequent losses of CH_3_OH and CO, respectively. These fragmentations are favored by the *ortho*-position of the phenolic OH relative to the methylated carboxy group (Fig. 1*B*). Moreover, the IR spectrum of methylated SA only shows a weak carbonyl (C=O) band at ṽ ∼1750 cm^−1^ which is characteristic of benzoic acid methyl esters (44). Instead, the main carbonyl absorption is shifted to ṽ = 1697 cm^−1^ due to the hydrogen bonding of the phenolic OH group to the C=O functionality of the *ortho*-positioned methyl ester group (Fig. 1*B*). This hydrogen bond also lowers the frequency of the phenolic O-H-bond from ∼3490 cm^−1^ to ∼3260 cm^−1^. Thus, both mass spectrometric and IR spectroscopic features reveal the *ortho*-positioning between the carboxy- and the phenolic OH-group in the benzol ring of the SA molecule.

We noted that the IR spectrum of analyte 1a is remarkably similar to the spectrum of methylated SA, with the frequencies of C=O and O-H-bonds at ṽ ∼1700 cm^−1^ and ∼3210 cm^−1^ suggesting a methylated 2-hydroxybenzoate (salicylate) structure (Fig. 1*C*). In addition, the IR spectrum of 1a reveals the presence of a formyl group in the molecule, as indicated by the occurrence of a characteristic pair of aldehydic C-H vibrations at ∼2810 cm^−1^ and ∼2725 cm^−1^ and a C=O vibration at ∼1730 cm^−1^. The latter overlaps with the methyl ester C=O absorbance at ∼1700 cm^−1^ but is discernible as a shoulder in the spectrum. Moreover, the mass spectrum of 1a contains an M^+^ ion at *m/z* 180, a main fragment ion at *m/z* 148, and an *m/z* 120 fragment, which indicates subsequent losses of CH_3_OH and CO from the M^+^ ion. An additional strong *m/z* 119 ion further indicates a loss of a formyl group from the main *m/z* 148 ion. Together, these spectroscopic data strongly suggested that 1a represents the methylated version of a formyl-salicylic acid isomer (Fig. 1*C*). A comparison of the GC-MS characteristic of the extract peak and authentic 5-formyl-salicylic acid finally established that the pathogen-inducible and ICS1-dependent compound 1 represents 5-formyl-SA (5-FSA) (Figs. 1*C*, and S1, *A* and *F*).

The IR spectra of analytes 2a and 3a also show the absorptions at ∼1700 cm^−1^ and ∼3210 cm^−1^ typical for hydrogen-bonded C=O methyl ester and OH functionalities, again suggesting methylated 2-hydroxybenzoate structures for both analytes (Fig. 1, *D* and *E*). The IR spectrum of 2a additionally contains a prominent carbonyl band at ∼ 1750 cm^−1^, indicating the presence of a second methyl ester group at the benzoate ring that is not involved in H-bonding. In the mass spectrum of 2a, an M^+^ at *m/z* 210 and fragment ions at *m/z* 178, 147, and 119 indicate subsequent losses of CH_3_OH, a methoxy (CH_3_O) moiety, and CO (Fig. 1*D*). Together, these MS and IR features were consistent with a carboxylated salicylic acid structure for 2a in its derivatized, bis-methylated form. Comparison with an authentic 5-carboxy-salicylic acid standard confirmed this assumption and showed that compound 2 represents 5-carboxy-SA (5-CSA) (Figs. 1*D*, and S1, *B* and *G*). In addition to the C=O vibration at ∼1700 cm^−1^, the IR spectrum of analyte 3a shows a second carbonyl absorption at 1760 cm^−1^ that is characteristic for C=O vibrations within methyl ester moieties attached to an aliphatic carbon (Fig. 1*E*; (44)). Moreover, the mass spectrum of 3a contains an M^+^ at *m/z* 224 which produces a fragment ion at *m/z* 192 by loss of MeOH. Both the M^+^ and *m/z* 192 can lose a fragment of *m/z* 59 which corresponds to a carboxymethyl (CO_2_CH_3_) group that can be released by α-cleavage from a methylated phenylacetic acid unit (Fig. 1*E*). Together, these spectroscopic data are consistent with a bis-methylated carboxymethyl-SA structure for 3a, and in analogy to the two other identified 5-substituted SA derivatives, we propose that compound 3 represents 5-carboxymethyl-SA (Fig. 1*E*).

The IR spectra of analytes 4a to 6a are all characterized by the absence of a phenolic OH-vibration but the presence of a methylated benzoate group, as indicated by the C=O vibration at 1747 cm^−1^ (Fig. 1, *F*–*H*). Similar to 1a, analyte 4a shows aldehydic C-H absorptions at ∼2810 cm^−1^ and ∼2725 cm^−1^ and a C=O vibration at 1725 cm^−1^, consistent with the presence of an additional formyl group in the molecule. The mass spectrum of 4a is characterised by an M^+^ of *m/z* 164 which is consistent with a formylated methylbenzoate structure. A main fragment ion at *m/z* 133 indicates cleavage of a methoxy group from the M^+^ ion. This dominant “CH_3_O” cleavage is characteristic of methylbenzoates and is also present in the mass spectra of 5a and 6a (Fig. 1, *F*–*H*), while the SA derivatives 1a to 3a preferentially show a loss of CH_3_OH due to their 2-hydroxy-methylbenzoate structure (Fig. 1, *B*–*D*). The loss of two CO units from the *m/z* 133 ion in the mass spectrum of analyte 4a corroborates its presumed formyl-benzoate structure. A comparison with the authentic substance confirmed that compound 4 is 3-formyl-benzoic acid (3-FBA) (Figs. 1*E*, and S1, *C* and *H*). Further, analyte 5a was identified as derivatized (*i.e.*, bis-methylated) 3-carboxybenzoic acid (3-CBA). Its IR spectrum shows a single, dominant C=O absorption at 1747 cm^−1^ caused by two chemically equivalent methylbenzoate carbonyls present in bis-methylated phthalate isomers. The M^+^ of *m/z* 194 and fragments corresponding to losses of a methoxy group (*m/z* 163) and subsequently CO (*m/z* 135) corroborated this phthalate structure. A comparison with the authentic substance then unequivocally identified compound 5 as 3-CBA (isophthalate) (Figs. 1*G*, and S1, *D* and *I*). Finally, the IR spectrum of analyte 6a shows a shoulder of the dominant methyl benzoate-associated C=O absorption (1747 cm^−1^) at ∼1760 cm^−1^, indicating, as for 3a, the presence of a methylated carboxyl group attached to an aliphatic moiety. The mass spectral characteristics (M^+^ of *m/z* 208, ion fragments of *m/z* 177 and *m/z* 149 indicating subsequent CH_3_O and CO fragment losses) thus strongly suggested that 6a is the derivatized (bis-methylated) form of 3-carboxymethyl benzoic acid (3-CMBA), in analogy with the accumulation of the proposed SA derivative 5-CMSA (3, Fig. 1*E*). Comparison with the available authentic compound confirmed that compound 6 represents 3-CMBA (Figs. 1*H*, and S1, *E* and *J*).

In summary, our analyses show that Arabidopsis leaves accumulate three 5-substituted SA derivatives carrying formyl, carboxy, and methylcarboxy units (5-FSA, 5-CSA, 5-CMSA), and the three analogues benzoic acid (BA) derivatives (3-FBA, 3-CBA, and 3-CMBA) upon *P. syringae* inoculation. Since the additional functionalities are located at the *meta*-position related to the carboxy group of SA or BA, we designate them as *meta*-substituted SA- and BA-derivatives. We were also able to detect the pathogen-induced accumulation of the six *meta*-substituted SA/BA-derivatives 1 to 6 by an inherently different analytical method that used liquid chromatography (LC) coupled with quadrupole time-of-flight mass spectrometry (qTOF-MS)-based high-resolution mass spectral analysis of underivatised Arabidopsis extracts (Fig. S2).

### Identification of the four remaining ICS1-dependent metabolites as SA-malate, SA-aspartate, N-pyruvoyl-glutamate, and isochorismoyl-glutamate

In the above-described GC-MS analysis, four other *Psm*-inducible and *ICS1*-dependent metabolites remained to be characterized (Fig. 1*A*). The mass spectrum of analyte 7a shows, just like SA methyl ester (Fig. 1*B*), the *m/z* 120 and *m/z* 92 fragment patterning typical for ester-linked SA derivatives (Fig. 2*A*). The readily detectable M^+^ ion showed a mass of *m/z* 282, and an ion at *m/z* 251 possibly representing an “M^+^-OCH_3_”-fragment was also discernible. On this basis, we hypothesized that analyte 7a represents the malate ester of SA (salicyloyl-malate; SA-Mal) in its derivatized, bis-methylated form. We next synthesized SA-Mal chemically (Fig. S3), and recorded the mass spectrum of the derivatized authentic substance. Its GC retention time and mass spectra were identical to those of analyte 7a, confirming that the plant-derived substance 7 is SA-Mal (Fig. 2, *A* and *B*). Further, exogenous application of a deuterated SA-form, D_4_-SA (in which the four hydrogens at the benzol ring are replaced by deuterium; Fig. S4), to *Psm*-inoculated leaves resulted in the formation of a product (D_4_-7a) that showed a mass spectrum consistent with a D_4_-SA-Mal structure, indicating *in planta* conversion of the applied D_4_-SA into D_4_-SA-Mal (Fig. 2, *C* and *D*). Therefore, Arabidopsis leaves synthesize the hitherto undescribed plant metabolite SA-Mal from SA in response to pathogen attack.

The mass spectrum of the derivatized compound 8a was identical to a published spectrum of bis-methylated salicyloyl-aspartate (SA-Asp) (Fig. 2*E*; (37)), indicating that analyte 8 represents SA-Asp. The M^+^ ion (*m/z* 281) of bis-methylated SA-Asp was readily detectable in the mass spectrum, and cleavage of the amide-bond resulted in a main salicyloyl-fragment at *m/z* 121 and a discernible *m/z* 160 ion representing the bis-methylated aspartyl-moiety. Supplementing *Psm*-inoculated leaves with exogenous D_4_-SA resulted in the generation of the corresponding D_4_-SA-Asp (D_4_-8a; Fig. 2*E*). The M^+^ of this compound fragmented into a main D_4_-salicyloyl-ion at *m/z* 125 and the remaining unlabeled aspartyl-associated fragment at *m/z* 160. In addition, plausible fragments derived from the aromatic molecule part by losses of methoxy or “CH_2_CO_2_” differed by 4 mass units in the unlabeled compound 8a and D_4_-labeled D_4_-8a, respectively (Fig. 2*E*). Together, this corroborates a *P. syringae*-inducible generation of SA-Asp from SA in Arabidopsis leaves. Notably, both SA-Mal and SA-Asp were also detected and characterised by LC-qTOF-MS-based, high-resolution mass spectral analysis of underivatised extracts (Fig. S2).

The IR spectrum of analyte 9a closely resembled the spectrum of a substance present in our IR spectral database, N-acetyl-glutamate dimethyl ester (Fig. 2*F*). This relates to the presence of an amide bond (N-H vibrations at ∼3400 cm^−1^, C=O absorption at ∼1710 cm^−1^, and N-C-vibrations at ∼1500 cm^−1^), and characteristic absorptions of a bis-methylated glutamate part (*e.g.*, strong absorptions at ∼1760 cm^−1^ of the 2 C=O vibrations of the methylester moieties). The IR spectrum of 9a therefore strongly suggested that a small organic acid similar to acetic acid is bound to glutamate *via* an amide linkage in compound 9. The mass spectrum of 9a contained a prominent ion at *m/z* 174 that was consistent with a (bis-methylated) glutamyl-fragment resulting from amide-cleavage of the presumed conjugate. This fragment could lose MeOH to generate the main ion at *m/z* 142 with a proposed pyrrolinone ring structure, and a further loss of MeOH would then produce the *m/z* 110 ion (Fig. 2*F*). When assuming pyruvate as the amide-linked conjugation partner for glutamate, both the *m/z* 202 (M^+^ - acetyl) and the *m/z* 214 (M^+^ - methoxy) could be plausibly explained as mass fragments of the derivatized form 9a. A bis-methylated *N*-pyruvate-glutamate (Pyr-Glu) structure would also be consistent with the IR spectrum of 9a, since the C=O absorption at ∼1760 cm^−1^ is overall more prominent than the same absorption of the database spectrum for *N*-acetyl-glutamate dimethylester, reflecting the presence of an additional ketonic C=O bond in pyruvate compared to acetate (Fig. 2*F*). Therefore, we propose that compound 9 represents *N*-pyruvoyl-glutamate (Pyr-Glu). Notably, in the course of our study, Pyr-Glu has been identified as side product in the ICS1- and PBS3-dependent biosynthesis of SA (21, 22), confirming our analysis. The same studies also identified isochorismoyl-glutamate (IC-Glu) as an intermediate in the isochorismate-derived biosynthesis of SA. The mass spectrum of 10a is consistent with a derivatized IC-Glu structure (Fig. 2*G*). On one hand, a m/z 379 ion represents a plausible M^+^-H_2_O ion fragment whose formation is favored because water loss of the six-membered ring in the not discernible M^+^ ion of derivatized (trimethylated) IC-Glu (*m/z* 397) would lead to a relatively stable benzolic fragment. On the other hand, the occurrence of the fragment pattern *m/z* 202, 174, 142, and 110, and other striking similarities with the mass spectrum of 9a in the medium and lower mass regions indicate the presence of the enolpyruvoylglutamyl part of the presumed IC-Glu (Fig. 2*G*). Therefore, our spectroscopic data also suggest the pathogen-induced, ICS1-dependent formation of both Pyr-Glu and IC-Glu as by-products and intermediates, respectively, of isochorismate-derived SA biosynthesis.

### SA functions as a precursor for SA-Mal and SA-Asp but not for the identified *meta-*substituted SA- and BA-derivatives; however, plants convert the respective formyl-into the carboxy-substituted compounds

**Figure 3:**
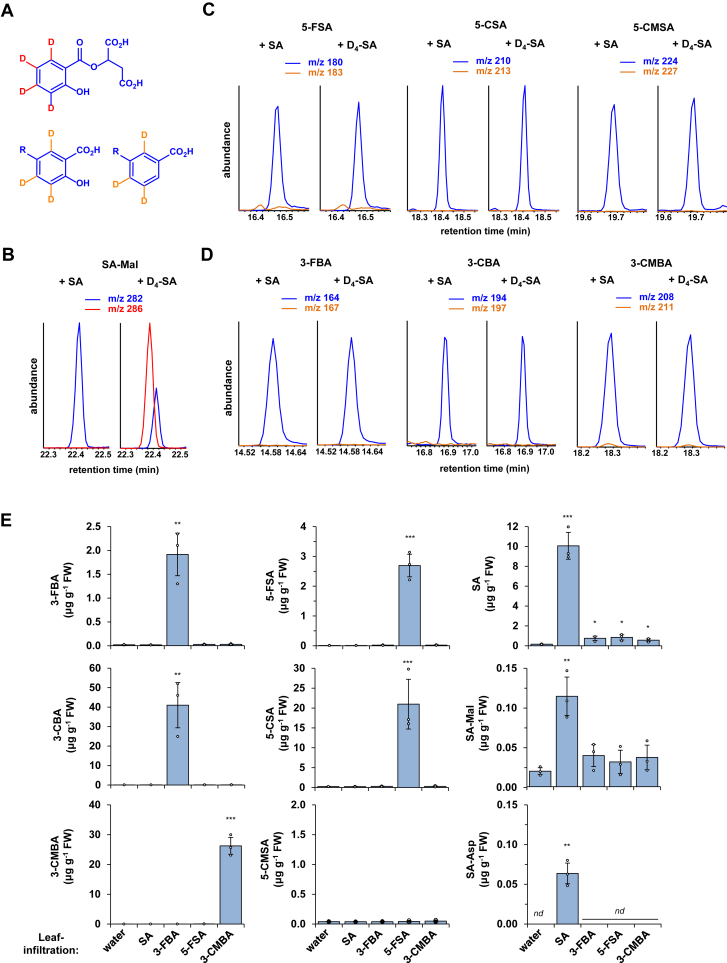
**In contrast to SA-Mal and SA-Asp, the six identified *meta*-substituted SA- and BA-derivatives are not biosynthesized from SA; however, the formyl-derivatives are oxidized to the respective carboxy compounds *in planta*.***A*–*D,* while exogenously supplemented SA is directly converted to SA-Mal (and SA-Asp, Fig. 2*E*) in leaves, it is not metabolized to *meta*-substituted SA and BA derivatives. *A*, theoretical deuteration patterns of SA-Mal, *meta*-substituted SAs and *meta*-substituted BAs when the metabolic conversion of D_4_-SA into these substances is assumed. This corresponds to shifts of 4 (D_4_-SA-Mal) and 3 (D_3_-labeled *meta*-substituted compounds) mass units compared to the unlabeled substances, respectively. *B*, exogenously supplemented D_4_-SA is metabolized to D_4_-SA-Mal. The depicted ion chromatograms correspond to *m/z* values of the M^+^ ions of SA-Mal (*blue*) and D_4_-SA-Mal (*red*) (Fig. 2, *A* and *C*). Chromatograms from extracts from leaves co-infiltrated with 0.5 mM SA and *Psm* (*left*) or 0.5 mM D_4_-SA and *Psm* (*right*) are shown. Leaves were harvested 48 h after infiltration. *B*, exogenously supplemented D_4_-SA is not converted to deuterium-labeled *meta*-substituted SA (*C*) or BA (*D*) derivatives *in planta*. The depicted ion chromatograms correspond to *m/z* values of the M^+^ ions (Fig. 1) of the *meta*-substituted compounds (*blue*) and of assumed, D_3_-labeled compounds (*orange*). Chromatograms from extracts from leaves co-infiltrated with 0.5 mM SA and *Psm* (*left panels*) or 0.5 mM D_4_-SA and *Psm* (*right panels*) are shown. Leaves were harvested 48 h after infiltration. *E*, feeding of Arabidopsis leaves with 3-formyl-BA and 5-formyl-SA provokes *in planta* formation of 3-carboxy-BA and 5-carboxy-SA, respectively, while exogenously supplemented SA is converted to SA-Mal and SA-Asp. Solutions of 0.5 mM of SA, 3-FBA, 5-FSA, or 3-CMBA or water as a control were infiltrated into three leaves of individual plants. The treated leaves were harvested 4 h later, and the levels of the three *meta*-substituted SA-derivatives (*left column*), the three *meta*-substituted BA-derivatives (*central column*), and SA, SA-Mal, and SA-Asp (*right column*) were analyzed by GC-MS. Bars represent the mean ± SD of three biological replicate samples, each consisting of six leaves from two plants. Individual data points of biological replicates are super-imposed on the bar graphs (*small circles*). The presence of *asterisks* above bars indicates significant differences between substance- and water-control treatment (∗∗∗*p* < 0.001, ∗∗*p* < 0.01, ∗*p* < 0.05; two-tailed *t* test).

Just as SA, the newly described *meta*-substituted SA- and BA-derivatives are generated in an ICS1-dependent manner (Fig. 1). To gain further insight into the formation of these compounds *in planta*, we tested whether SA would function as their biosynthetic precursor. To do so, we co-infiltrated Arabidopsis leaves with 0.5 mM D_4_-SA and *Psm* and examined the possible formation of labeled compounds by GC-MS analyses 48 h later (Fig. 3, *A*–*D*). In theory, the *meta*-substituted compounds should lose one deuterium at the aromatic ring during their formation from D_4_-SA to produce D_3_-labeled variants, while D_4_-labeled variants of SA-Mal and SA-Asp should be formed (Fig. 3*A*). When ion chromatograms corresponding to masses of M^+^ ions or of the main aromatic fragments at *m/z* 120 (SA-Mal) and *m/z* 121 (SA-Asp) were compared with the corresponding (*m* + 4)-chromatograms, the *in planta* formation of both D_4_-SA-Mal and D_4_-SA-Asp was evident in addition to their unlabeled counterparts (Figs. 2, *C*–*E*, and 3*B*), indicating that SA functions as a biosynthetic precursor for SA-Mal and SA-Asp. However, overlays of chromatograms corresponding to the masses of M^+^ ions and the respective (*m* + 3)-masses only showed accumulation of the natural compounds, while deuterium-labeled versions were not generated (Fig. 3, *C* and *D*). This shows that SA does not function as a precursor for the biosynthesis of the *meta*-substituted aromatics 1 to 6.

To further examine the biosynthesis of the *meta*-substituted SA- and BA-derivatives, we infiltrated the leaves of plants with 0.5 mM solutions of SA, 3-FBA, 5-FSA, or 3-CMBA and assayed metabolite contents 4 h later (Fig. 3*E*). As expected, exogenous application of each of the compounds resulted in increases in their contents in the treated leaves. Remarkably, the application of 3-FBA and 5-FSA resulted in a strong accumulation of 3-CBA and 5-CSA, respectively, indicating efficient conversion of the formyl-substituted BA- and SA-derivatives into their carboxy-substituted counterparts *in planta*. This indicates the presence of oxidoreductase-like enzyme activities in the leaves that catalyze the oxidation of the BA- and SA-aldehydes into the respective carboxylic acids (Fig. 3*E*). By contrast, 3-CMBA was not converted to any of the other *meta*-substituted compounds. However, exogenous SA increased the levels of both SA-Mal and SA-Asp, corroborating that these conjugates are synthesized from SA. Interestingly, the application of 3-FBA, 5-FSA, and 3-CMBA also modestly increased the leaf levels of SA, which might indicate that treatments with these compounds stimulate SA biosynthesis to a small extent (Fig. 3*E*).

### While isochorismate-derived metabolism is negatively regulated by NPR1, SA conjugates, and *meta*-substituted SA/BA-derivatives are oppositely affected by PBS3

**Figure 4:**
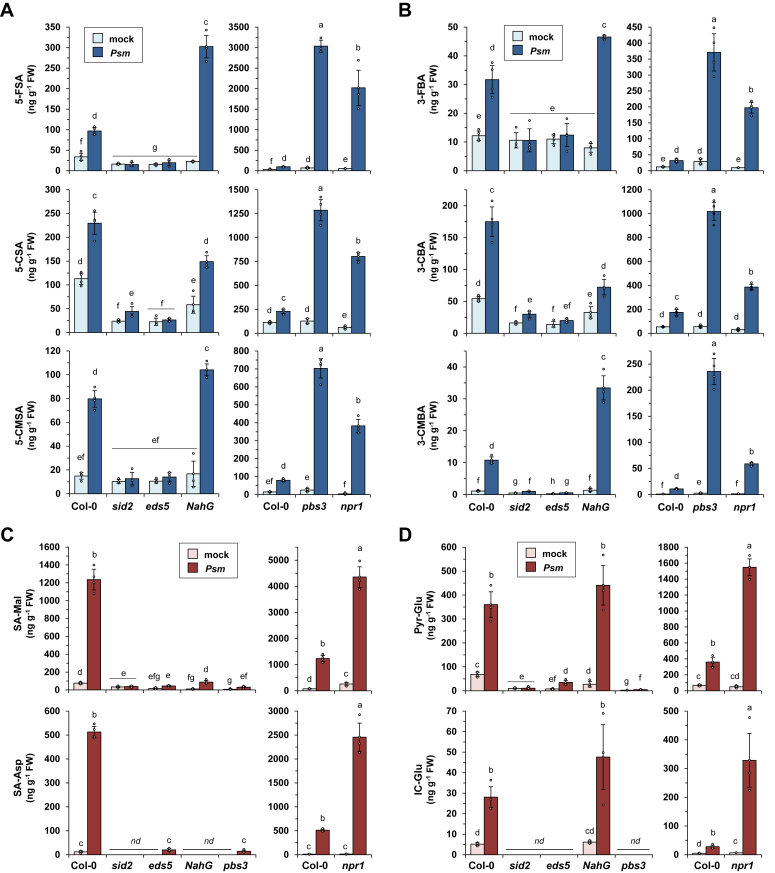
***meta*-Substituted SA/BA-derivatives and SA-conjugates show similar accumulation characteristics in the SA pathway mutants *sid2*, *eds5*, and *npr1* but are oppositely regulated by *PBS3*.***A* and *B*, *meta*-Substituted SA- (*A*) and BA-derivatives (*B*) show compromised pathogen-induced accumulation in *sid2* and *eds5* mutants but not in SA degrading NahG plants, and they over-accumulate in both pathogen-inoculated *pbs3* and *npr1. A,* levels of 5-FSA, 5-CSA, and 5-CMSA in the leaves of Col-0, *sid2*, *eds5*, NahG, *pbs3*, and *npr1* plants 48 h after inoculation with *Psm* (*dark blue*) or mock-control infiltration (*light blue*). Metabolite levels are given in ng per gram fresh weight (FW). *B,* levels of 3-FBA, 3-CBA, and 3-CMBA (details as in *A*). *C*, SA-Mal and SA-Asp exhibit diminished accumulation in all the SA biosynthetic mutants and in SA catabolizing NahG plants, and they over-accumulate in *npr1*. Metabolite levels (ng per gram FW) in *Psm*- (*dark red*) and mock-inoculated (*light red*) leaves at 48 h post treatment (hpt) are given. *D*, Pyr-Glu and IC-Glu are biosynthesised in dependence of *ICS1/SID2*, *EDS5*, and *PBS3*, accumulate in NahG and over-accumulate in *npr1*. Metabolite levels (ng per gram FW) in *Psm*- (*dark red*) and mock-inoculated (*light red*) leaves at 48 hpt are given. For (*A*–*D*), the bars represent the mean ± SD of four biological replicates from different plants, each replicate consisting of six leaves from two plants (nd: not detected). Individual data points of biological replicates are super-imposed on the bar graphs (*small circles*). Different letters above the bars indicate statistically significant differences (*p* < 0.05, Kruskal-Wallis H test). Please note that the data for the Col-0 wildtype are depicted twice in the figures with different scaling of the y-axes to better illustrate comparisons with moderately accumulating (*left panels*) and over-accumulating (*right panels*) lines.

To investigate the regulatory principles by which the ten identified ICS1-dependent metabolites accumulate (Figs. 1 and 2), we compared their *Psm*-induced generation at 48 h after inoculation in the leaves of the Col-0 wildtype, the three SA biosynthetic mutants *sid2*, *eds5*, and *pbs3* (18, 19, 23, 45), the NahG transgenic line that rapidly degrades SA due to expression of an SA hydroxylase (46), and in the *npr1* mutant defective in SA perception and downstream responses (47) (Fig. 4). Remarkably, all six *meta*-substituted SA/BA-derivatives showed a compromised *Psm*-triggered accumulation in *sid2* and *eds5*, while they were strongly over-produced upon pathogen inoculation in *pbs3* and *npr1* (Fig. 4, *A* and *B*). This indicates that ICS1 and the putative isochorismate-transporter EDS5 are required for their biosynthesis, while PBS3 and NPR1 strongly dampen their accumulation. Further, NahG plants that promptly degrade biosynthesized SA to catechol (48) show a significant accumulation of *meta*-substituted SA/BA-derivatives, corroborating our conclusion that these compounds are not derived from SA (Fig. 4, *A* and *B*). Compared to the wildtype, NahG showed enhanced accumulation of the formyl- (5-FSA, 3-FBA) and carboxymethyl- (5-CMSA, 3-CMBA) compounds, and attenuated accumulation of the *meta*-carboxylated substances (5-CSA, 3-CBA).

By comparison, the *Psm*-triggered accumulation of the SA conjugates SA-Mal and SA-Asp was compromised in all of the SA biosynthetic mutants as well as in the NahG line, confirming their derivation from SA and illustrating that the whole SA biosynthetic pathway is required for their biosynthesis (Fig. 4*C*). Moreover, Pyr-Glu and IC-Glu, the by-product and intermediate of SA biosynthesis, respectively, exhibited compromised accumulation in the SA biosynthetic mutants *sid2*, *eds5* and *pbs3*, but were generated in a wildtype-like manner in the NahG transgenics (Fig. 4*D*).

### *Meta*-substituted SA/BA-derivatives show a more moderate accumulation pattern than other ICS1-derived metabolites in *P. syringae*-inoculated leaves

**Figure 5:**
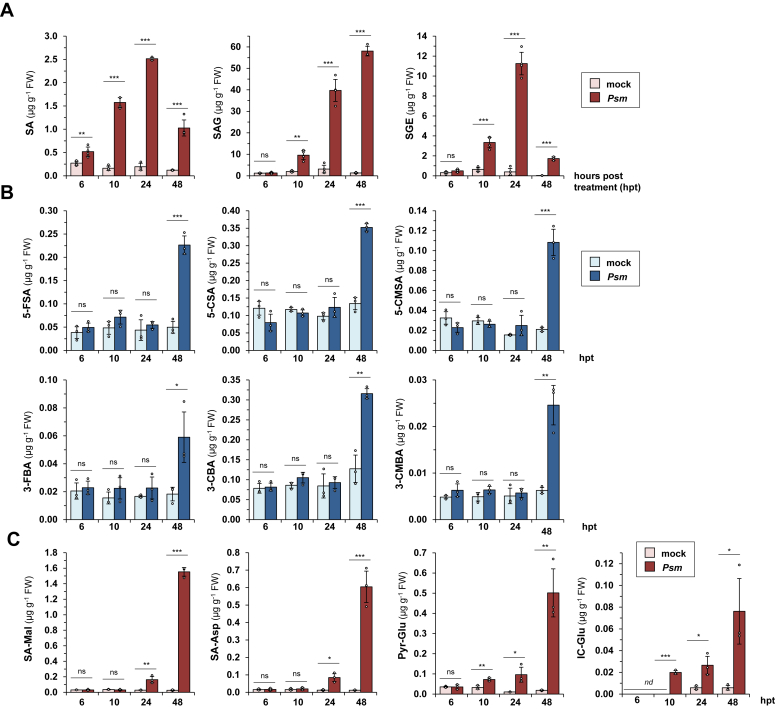
***Meta-s*ubstituted SA/BA-derivatives accumulate later and to more moderate levels in *P. syringae*-inoculated leaves than other detected ICS1-dependent metabolites.***A*–*C*, levels of different *ICS1*-dependent metabolites at 6, 10, 24, and 48 h post-treatment in mock-infiltrated (mock) and *Psm*-inoculated (*Psm*) leaves of Arabidopsis Col-0 plants. Three rosette leaves per plant were treated. Metabolite contents were determined by GC-MS analysis and are given in μg per gram FW. Bars represent means ± SD of three to four biological replicates. One replicate sample consisted of six leaves from two plants. Individual data points of biological replicates are super-imposed on the bar graphs (*small circles*). The presence of asterisks above the lines indicates whether significant differences between mock- and *Psm*-treatment exist for the particular timepoint (∗∗∗*p* < 0.001, ∗∗*p* < 0.01, ∗*p* < 0.05; ns: no significant differences; two-tailed *t* test). *A*, SA, SA-ß-glucoside (SAG), and SA glucose ester (SGE). *B*, *meta*-substituted SA/BA-derivatives: 5-FSA, 5-CSA, 5-CMSA, 3-FBA, 3-CBA, and 3-CMBA. *C*, SA-Mal, SA-Asp, Pyr-Glu, and IC-Glu.

To compare the kinetics and degree of pathogen-induced accumulation of the different ICS1-dependent substances in Arabidopsis, we *Psm*-inoculated or mock-treated leaves of Col-0 plants, harvested the treated leaves at 6, 10, 24, and 48 h after the treatments, and assessed their metabolite contents *via* GC-MS-based analyses (Fig. 5). Remarkably, SA showed the fastest *Psm*-induced increase of all of the examined metabolites (Fig. 5*A*). It significantly accumulated already at 6 h post-inoculation (hpi) with *Psm*, and its levels gradually increased to a maximum at 24 hpi and then decreased again at 48 hpi. By contrast, the SA glucose conjugates SAG and SGE accumulated later than 6 hpi, with a first significant pathogen-induced increase at 10 hpi. The accumulation pattern of the glucose ester SGE thereby paralleled the pattern for free SA, with a distinct maximum accumulation at 24 hpi. By comparison, the glucoside SAG showed a steady and strong increase until 48 hpi (Fig. 5*A*). Quantitatively, SAG showed the most prominent increase after inoculation (>50 μg g^−1^ FW at 48 hpi), followed by SGE (>10 μg g^−1^ FW at 24 hpi) and SA (∼2.5 μg g^−1^ FW at 24 hpi), which is consistent with the notion that SAG represents the most prominent SA derivative in Arabidopsis (9).

The temporal accumulation patterns of Pyr-Glu and IC-Glu were similar to those of SAG, but their quantitative levels were much more moderate (by far lower than 1 μg g^−1^ FW; Fig. 5*C*). Moreover, SA-Mal and SA-Asp did not accumulate before 24 hpi and showed the most prominent pathogen-induced increases at 48 hpi, indicating that both SA conjugates start to accumulate later than the glucose derivatives SAG and SGE (Fig. 5*C*). At 48 hpi, the absolute levels of SA-Mal and SA-Asp were on a similar scale than those of free SA (∼1–2 μg g^−1^ FW). Finally, the six *meta*-substituted substances 5-FSA, 5-CSA, 5-CMSA, 3-FBA, 3-CBA, and 3-CMBA showed a similar pattern of accumulation, with increased levels of these compounds detected not earlier than 48 hpi (Figs. 5*B*, and S5*A*). Moreover, the absolute levels of each of the six metabolites were consistently lower than 0.5 μg g^−1^ FW. Together, this indicates that the accumulation of the six *meta*-substituted SA/BA-derivatives in the leaves starts at relatively late times after *P. syringae*-inoculation and results in comparatively moderate quantitative increases of these substances.

In addition to Col-0 leaves inoculated by the compatible, virulent *Psm* strain, the six *meta*-substituted SA/BAs and the other investigated four SA-related metabolites also accumulated in leaves inoculated with an avirulent, hypersensitive-response-inducing *Psm* strain expressing the *AvrRpm1* avirulence gene (*Psm avrRpm1*; (34)) (Fig. S5). Notably, five of the six meta-substituted SA/BAs started to accumulate earlier in the incompatible *Psm avrRpm1-*Col-0 interaction than in the compatible *Psm-*Col-0 interaction (Fig. S5*A*).

### Administration assays suggest that *meta*-substituted SA/BA-derivatives and SA-Mal have modest activity in the induction of acquired resistance and *PR1* gene expression

**Figure 6:**
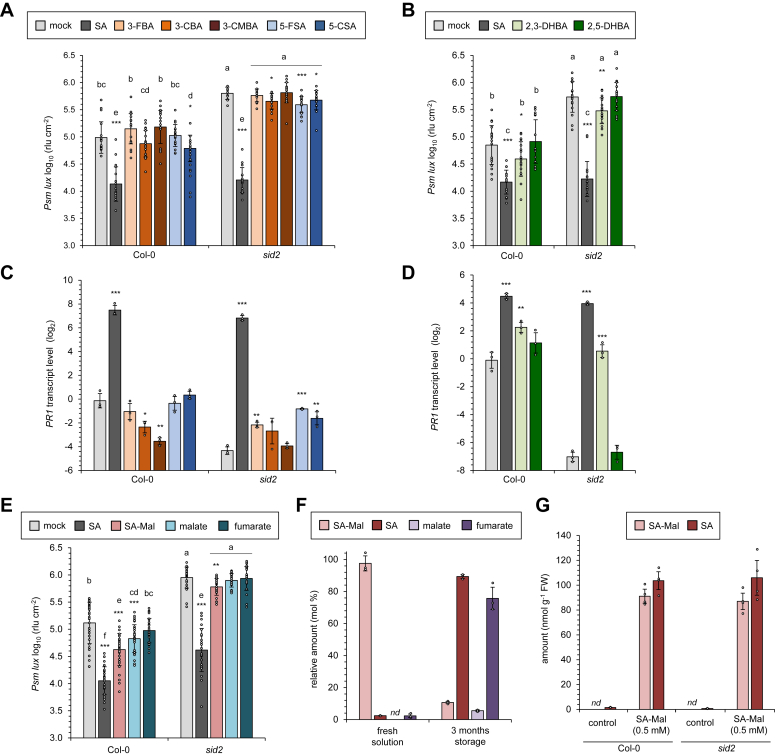
**Exogenously applied, *meta*-substituted SA/BA-derivatives and SA-malate have moderate impact on plant immunity and pathogenesis-related gene expression.***A* and *B*, assessment of resistance induction by exogenous application of SA and different SA- and BA-derivatives. *A*, application of SA, 3-FBA, 3-CBA, 3-CMBA, 5-FSA, or 5-CSA. *B*, application of SA, 2,3-dihydroxybenzoic acid (2,3-DHBA), or 2,5-dihydroxybenzoic acid (2,5-DHBA). *A* and *B*, three leaves of individual Arabidopsis wild-type (Col-0) or *sid2* mutant plants were infiltrated with a 0.5 mM substance solution. Control plants (mock) were infiltrated with water containing 0.1% ethanol, the medium in which the applied substances were dissolved. The three pre-treated leaves were challenge-inoculated 4 h later with a suspension of bioluminescent *P. syringae* pv. *maculicola* ES4326 *(Psm)* strain expressing the *luxCDABE* operon from *Photorhabdus luminescens* (*Psm lux*; (92)) by syringe-infiltration (OD_600_ = 0.001). As a measure of plant susceptibility, the numbers of bacteria were determined at 60 h post-inoculation (hpi) in inoculated leaves by luminescence quantification and expressed as relative light units (rlu) per cm^2^ leaf area (93). The data values were log_10_-transformed for graphical display. Bars indicate the mean ± SD of at least 15 leaf replicates. Different letters denote significant differences (*p* < 0.05) according to ANOVA and *post hoc* Tukey HSD test. Moreover, the results of two-tailed *t*-tests between the mock- and the treatment-samples of one genotype are indicated by the presence/absence of asterisks above the bars of the treatment samples (∗∗∗*p* < 0.001, ∗∗*p* < 0.01, ∗*p* < 0.05; absence of asterisks: no significant differences). *C* and *D*, assessment of inducible expression of the defence gene *PATHOGENESIS-RELATED1* (*PR1*) by exogenous SA and different SA- and BA-derivatives. *C*, application of SA, 3-FSA, 3-BSA, 3-CMBA, 5-FSA, or 5-CSA. *D*, application of SA, 2,3-DHBA, or 2,5-DHBA. *C* and *D*, three leaves of individual Col-0 or *sid2* plants were infiltrated with a 0.5 mM substance solution or with water containing 0.1% EtOH as a control treatment (mock). The treated leaves were harvested 4 h later, and transcript levels of *PR1* were determined by RT-qPCR using *POLYPYRIMIDINE TRACT-BINDING PROTEIN 1* (*PTB1*) as the reference gene (Fig. S7). Bars represent means ± SD of gene transcript levels calculated from the values of the three biological replicates, which themselves represented the mean of two technical replicates. The data values were log_2_-transformed for graphical display. The presence of asterisks above the bars indicates whether significant differences between mock- and substance-treatments exist for a particular genotype (∗∗∗*p* < 0.001, ∗∗*p* < 0.01, ∗*p* < 0.05; absence of asterisks: no significant differences; two-tailed *t* test). *E*, assessment of resistance induction of SA-malate and related compounds. Three leaves of individual Col-0 or *sid2* mutant plants were infiltrated with 0.5 mM solutions of SA, SA-Mal, malate, or fumarate. A mock-control infiltration was performed with water containing 0.1% EtOH. The three pre-treated leaves were challenge-inoculated 4 h later with *Psm lux*, and bacterial numbers were assessed after 60 h as described above. The values were log_10_-transformed for graphical display. Bars indicate the mean ± SD of at least 24 leaf replicates. Statistical analyses as described for (*A* and *B*). *F*, storage of SA-malate solutions over longer time periods resulted in degradation to SA, malate, and fumarate. A solution of 100 ng μl^−1^ SA-Mal in MeOH/H_2_O (80:20) was freshly prepared, and 10 μl thereof was analyzed by GC-MS the same day (*left*) or 3 months later (*right*). The assessed relative contents of SA-Mal, SA, malate, and fumarate (related to the amount of employed SA-Mal) are given. *G*, SA-Malate exogenously supplemented to leaves is hydrolyzed to SA. A solution of 0.5 mM SA-Mal or water containing 0.1% EtOH (control) was infiltrated into three leaves of individual Col-0 plants, and the treated leaves were harvested 48 h later. The contents of SA-Mal and SA in leaves were assessed and expressed in nmol g^−1^ FW. *A*–*G*, individual data points of biological (*F*, technical) replicates are super-imposed on the bar graphs (*small circles*).

We next asked whether the *in-planta-*detected *meta*-substituted SA/BA-derivatives and SA-Mal would exert an impact on plant immunity and defense-related gene expression. It is well established that plants exogenously supplemented with SA acquire resistance to a series of compatible pathogens and increase expression of the SA marker gene *PATHOGENESIS-RELATED1* (*PR1*) (46, 49, 50). Consistently, infiltration of a 0.5 mM solution of SA into the leaves of Arabidopsis Col-0 plants robustly increased the resistance of these leaves to subsequent infection by *Psm* in different experiments, and bacterial growth after inoculation was reduced by about one order of magnitude (Fig. 6, *A*, *B*, and *E*). Moreover, the ICS1-defective *sid2* mutant allowed a significantly higher bacterial growth in control plants than Col-0, indicating that the inability to induce SA biosynthesis in the mutant compromises plant basal resistance (Fig. 6, *A*, *B*, and *E*; (18, 19)). The treatment of *sid2* with exogenous SA resulted in a strong acquired resistance response, by which bacterial growth in the course of pathogen challenge was attenuated by nearly two orders of magnitude (Fig. 6, *A*, *B*, and *E*). As a consequence, SA-treated *sid2* acquired similar (Fig. 6, *A* and *B*) or only modestly lower (Fig. 6*E*) levels of resistance than SA-induced Col-0 plants.

When plants were treated with 0.5 mM solutions of the five *meta*-substituted SA/BA derivatives for which authentic substances were available, we observed much weaker immune responses than for SA administration (Fig. 6*A*). Col-0 plants lacked resistance induction towards *Psm* after treatment with 3-FBA, 3-CBA, 3-CMBA, and 5-FSA, and only 5-CSA induced a modest elevation of pathogen resistance in Col-0. Exogenous 5-CSA also showed a modest positive effect on the resistance of *sid2*, and the same was true for 3-CBA and 5-FSA applications (Fig. 6*A*). Comparatively, we also examined the ability of resistance induction for two other known SA derivatives, 2,3-dihydroxybenzoic acid (2,3-DHBA), and 2,5-DHBA, which occur in Arabidopsis leaves primarily as glycosylated variants (30, 32). Similar to most of the here-identified *meta*-substituted SA/BA-derivatives, a 0.5 mM solution of 2,5-DHBA showed no effect on plant resistance to *Psm* infection (Fig. 6*B*). However, exogenously applied 2,3-DHBA increased immunity to a moderate extent in both Col-0 and *sid2*, but again, this response was significantly weaker than the immune responses towards the same dose of SA (Fig. 6*B*).

**Figure 7:**
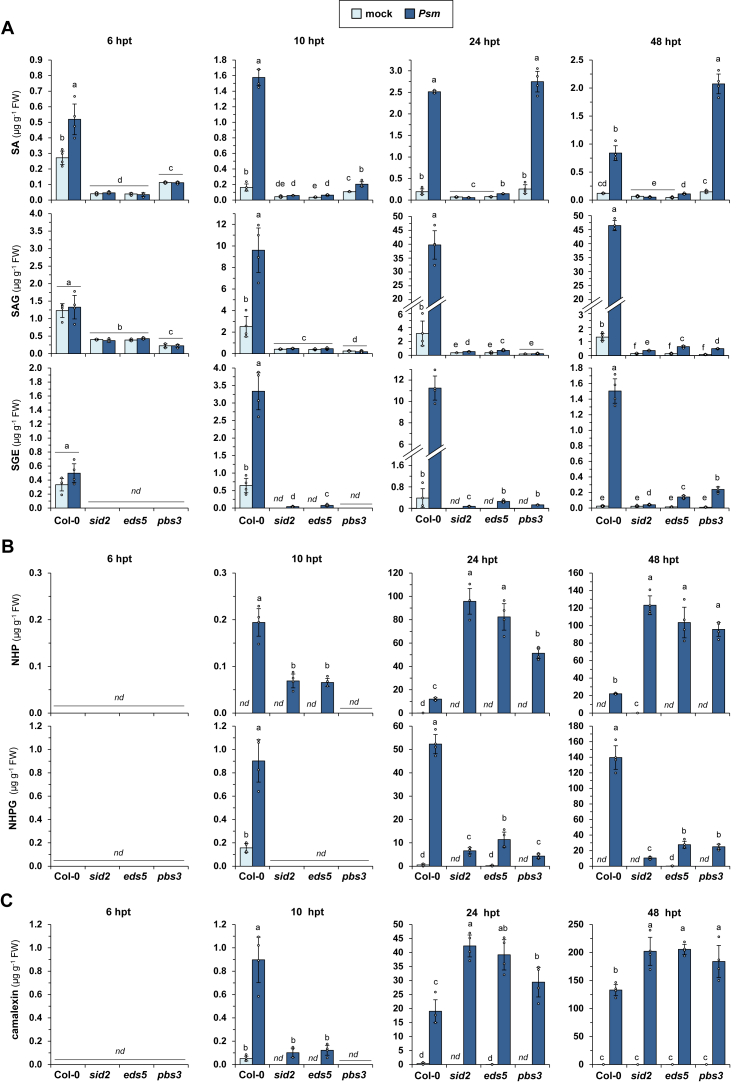
**While an *ICS1*-dependent but *PBS3*-independent biosynthetic branch leads to the *Psm*-induced accumulation of free salicylic acid at later infection stages, *N*-hydroxypipecolic acid over-accumulates to similar levels in *sid2*, *eds5*, and *pbs3*.***A*–*C*, levels of different defense-related metabolites at 6, 10, 24, and 48 h post-treatment (hpt) in mock-infiltrated (mock) and *Psm*-inoculated (*Psm*) leaves of Arabidopsis Col-0, *sid2*, *eds5*, and *pbs3* plants. Three rosette leaves per plant were treated. Metabolite contents were determined by GC-MS analysis of trimethylsilylated analytes and are given in μg per gram FW. Bars represent means ± SD of four biological replicates. One replicate sample consisted of six leaves from two plants. Individual data points of biological replicates are super-imposed on the bar graphs (*small circles*). Different letters denote significant differences (*p* < 0.05, Kruskal-Wallis H test). *A*, levels of SA, SAG, and SGE. *B*, levels of NHP and NHP-glucoside (NHPG). *C*, levels of camalexin. See also Fig. S6.

Next, we assessed the transcript levels of *PR1* in the leaves of mock-treated control plants and of plants treated with SA and the above-described SA-related substances (Fig. 6, *C* and *D*). Remarkably, the basal transcript levels of *PR1* in the control plants were significantly lower in *sid2* than in Col-0 (Fig. 6, *C* and *D*), which correlated with the basal endogenous contents of SA and the degree of basal resistance in these plants (Figs. 6, *A*, *B*, and *E*; Fig. 7*A*). As expected, exogenous SA triggered a strong elevation of the transcript levels of *PR1* in the treated leaves of both Col-0 and *sid2* (Fig. 6, *C* and *D*). By contrast, none of the five applied *meta*-substituted SA/BA-derivatives was able to increase *PR1* transcript levels in the Col-0 wildtype. Upon 3-CMBA and 3-CBA application, the basal *PR1* levels were even significantly decreased in Col-0. For 3-CMBA-treated plants, this negative effect on *PR1* expression correlated with a (statistically non-significant) tendency of increased susceptibility towards *Psm* infection (Fig. 6*A*). Notably, 3-FBA, 5-FSA, and 5-CSA moderately increased the low basal levels of *PR1* transcripts of *sid2*, while 3-CMBA and 3-CBA exerted no significant effects in the mutant (Fig. 6*C*). Together with the resistance data, this indicates that certain *meta*-substituted SA/BA-derivatives (in particular 5-CSA and 5-FSA) can moderately elevate immune responses in the SA-deficient *sid2* plants that exhibit strongly reduced basal defenses. In this context, we found that application of 2,3-DHBA, but not 2,5-DHBA, markedly elevated *PR1* transcript levels (Fig. 6*D*), which again paralleled the effects of the two DHBAs on acquired resistance (Fig. 6*B*).

Finally, we infiltrated 0.5 mM solutions of chemically synthesized SA-Mal to Col-0 and *sid2* plant and scored resistance to *Psm* challenge in the treated leaves (Fig. 6*E*). Significant induction of resistance was observed in both Col-0 and *sid2* plants upon SA-Mal application, but this effect was modest compared to the effect of exogenous SA. Moreover, we found that in solution, SA-Mal is slowly decomposed to SA, malate, and fumarate. In a freshly prepared solution that underwent the analytical workup process and subsequent GC-MS analysis on the same day, about 97% of the initially dissolved SA-Mal, but also 3% of free SA and fumarate were detectable in the analysis (Fig. 6*F*). After 3 months of storage of the solution at 4 °C, about 90% of the SA-Mal was decomposed to free SA, and significant amounts of fumarate and malate were also detected in the solution (Fig. 6*F*). Therefore, we also tested whether 0.5 mM solutions of fumarate or malate would affect pathogen resistance, and found a small resistance increase for malate but not for fumarate application (Fig. 6*E*). Moreover, when infiltrating fresh SA-Mal solution into Arabidopsis Col-0 or *sid2* leaves, about 50% of the SA-Mal was hydrolyzed to free SA in the leaves after 2 days (Fig. 6*G*). Considering this efficient *in planta* release of SA from SA-Mal, and the strong immune-stimulating activity of SA, we consider it as likely that the moderate resistance effects observed for exogenous SA-Mal application were mainly caused by the liberated SA.

Together, our administration assays suggest that the *meta*-substituted SA/BA-derivatives exert, when compared to SA, only modest immune-stimulatory activity in Arabidopsis, which was most evident in the SA-deficient *sid2* mutant. Out of the five tested substances, 5-CSA and 5-FSA showed the most pronounced stimulatory effects. SA-Mal application also resulted in a significant resistance effect, but this is supposedly a result of a release of SA from SA-Mal *in planta*. In addition, our results indicate that 2,3-DHBA but not 2,5-DHBA has moderate immune-activating activity.

### An *ICS1*-derived but *PBS3*-independent accumulation of unconjugated SA occurs in the later stages of the Arabidopsis-Pseudomonas interaction

According to a recently established model, the isochorismate pathway-derived, stress-inducible biosynthesis of SA in Arabidopsis proceeds *via* the ICS1-catalysed conversion of chorismate to isochorismate in the plastid, a translocation of isochorismate into the cytosol by the MATE transporter EDS5, and conjugation of isochorismate to Glu by cytosolic PBS3. The resulting intermediate IC-Glu would then be transformed to SA *via* the release of Pyr-Glu (21, 22). Knockout mutants of the isochorismate pathway genes *ICS1*, *EDS5*, and *PBS3* should therefore be severely compromised in the biotic stress-inducible accumulation of SA and its conjugates. Contrasting this assumption, our metabolite analyses indicated a significant accumulation of unconjugated SA in the leaf samples of *pbs3* knockout plants. At 48 h post-inoculation with *Psm, pbs3* even over-accumulated SA, and this tendency was confirmed by two inherently different methodological procedures that used GC-MS-based and LC-qTOF-MS-based analyses, respectively (Figs. 7*A*, and S2*B*). This finding prompted us to include the main SA biosynthetic mutants *sid2* (*ics1*), *eds5*, and *pbs3* in our time-course analysis, in which metabolite accumulation was assessed in the leaves of *Psm-*inoculated and mock-treated plants at 6, 10, 24 and 48 hpi (Fig. 7).

This analysis showed that *sid2* mutants failed to accumulate free SA in response to *Psm* over the whole time period between 6 and 48 hpi (Fig. 7*A*). In addition, the amounts of the two main conjugates, SAG and SGE, which were synthesized in the Col-0 wildtype to high levels from 10 hpi onwards, only faintly increased in *sid2* at 24 and 48 hpi to levels that were well below the basal contents of the unstressed wildtype (Fig. 7*A*). For a quantitative comparison, we approximated the sum of SA, SAG, and SGE as the levels of “total SA”. The increases of total SA in *sid2* following *Psm* inoculation only amounted to ∼0.5% of the corresponding increases in Col-0 at 48 hpi, the latest investigated timepoint for which the pathogen responses were highest. This indicates that virtually the whole metabolic flow towards biosynthesis of SA and its main derivatives under biotic stress proceeds *via* ICS1. The biotic-stress-induced biosynthesis of SA was also greatly reduced in *eds5*, for which marginal *Psm*-induced accumulation of SA, SAG, and SGE was detected at 24 and 48 hpi but not at earlier stages (Fig. 7*A*). For *eds5*, the increases of the total SA levels at 48 hpi amounted to ∼1.5% of the respective increases in the wildtype, indicating that ICS1-mediated SA biosynthesis is almost fully carried forward by EDS5. Although the *Psm*-inducible accumulation of free SA was heavily compromised in *pbs3* during the early stages of infection (6 and 10 hpi), SA started to accumulate in this mutant at 10 hpi, reached wild-type-like accumulation levels at 24 hpi, and even over-accumulated SA at 48 hpi. However, as for *sid2* and *eds5*, *pbs3* only weakly accumulated the glucose conjugates SAG and SGE over the whole time period (Fig. 7*A*). In addition, as described above, the same stringent dependency on *ICS1*, *EDS5*, and *PBS3* was observed for the two other SA conjugates, SA-Mal and SA-Asp (Fig. 4*C*). According to the above-applied sample calculation, *pbs3* accumulated about 5.5% of the “total SA” accumulating in Col-0 at 48 hpi, and the main part of this overall moderate increase was due to the accumulation of free, unconjugated SA (Fig. 7*A*). In summary, this indicates that in addition to a major branch of the isochorismate pathway that includes ICS1, EDS5, and PBS3, a second metabolic branch exists that proceeds independent of PBS3, becomes relevant at later stages during the plant-pathogen encounter, and almost exclusively results in the production of unconjugated SA. It is noteworthy that the described tendencies were congruently detected by both the GC-MS- and LC-qTOF-MS-based analytic procedures (Figs. 7*A*, and S2*B*).

### The *sid2*, *eds5*, and *pbs3* mutants show similar immune-related and metabolic characteristics

**Figure 8:**
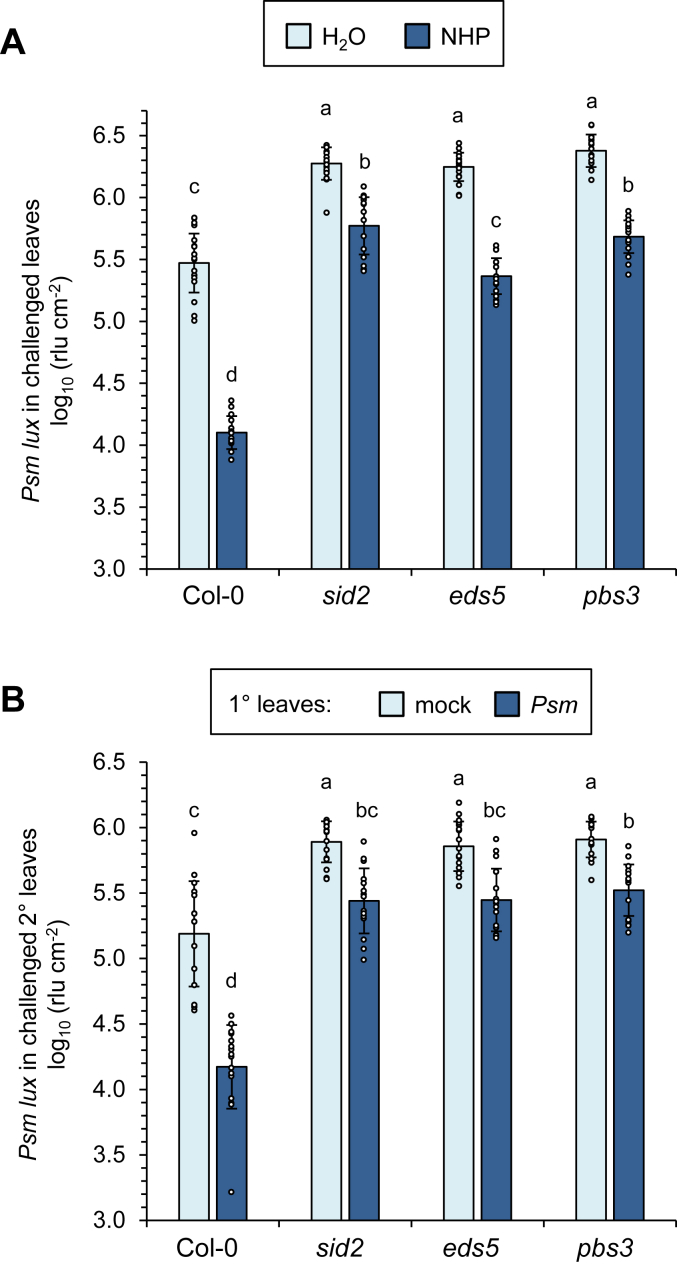
**The establishment of systemic acquired resistance by exogenous NHP or as a consequence of an inducing pathogen inoculation is compromised to similar degrees in *sid2*, *eds5*, and *pbs3*.***A*, SAR induced by exogenous NHP treatment is attenuated in the SA biosynthetic mutants *sid2*, *eds5*, and *pbs3*. The soil substrate of individually cultivated Arabidopsis Col-0, *sid2*, *eds5*, or *pbs3* plants was supplemented with 10 ml of a 1 mM NHP solution or 10 ml of water. One day later, three rosette leaves were inoculated with *Psm lux*. Bacterial numbers in the challenged leaves were assessed at 60 dpi (see Fig. 6). Bars indicate the mean ± SD of at least 15 replicate leaf samples. *B*, *P. syringae*-triggered SAR is diminished in all the SA biosynthetic mutants to a similar extent. To assess SAR establishment, three lower (1°) leaves per plant (Col-0, *sid2*, *eds5*, or *pbs3*) were either inoculated with *Psm* or mock-infiltrated with 10 mM MgCl_2_. Two days later, three upper (2°) leaves were challenge-inoculated with *Psm lux*, and bacterial numbers in the 2° leaves scored 60 h after the challenge-inoculation. Bars show the mean ± SD of at least 18 leaf replicates. *A* and *B*, individual data points of biological replicates are super-imposed on the bar graphs (*small circles*). Different letters denote significant differences (*p* < 0.05, ANOVA and *post hoc* Tukey HSD test).

We and others have previously described several immune-related characteristics of the ICS1-defective *sid2* mutant that were explained by its SA-deficient metabolic phenotype. First, *sid2* shows an attenuated accumulation of the SAR inducer *N*-hydroxypipecolic acid (NHP), its biosynthetic precursor pipecolic acid (Pip), and the phytoalexin camalexin in the early (10 hpi) interaction stages with *Psm* (12). Second, *sid2* over-accumulates NHP, its glucose ester NHPGE, and camalexin at later infection stages (24 and 48 hpi) (10, 18, 26, 50). Third, *sid2* shows strongly attenuated accumulation of the NHP *N*-*O*-glucoside NHPG (26, 50). And finally, *sid2* shows a significantly attenuated but not fully compromised SAR response, a tendency that was observed for both the biological induction of SAR following a localized *Psm* inoculation (12, 49, 50), and for chemical induction of SAR by pre-treatment of plants with exogenous NHP (10, 11). We wondered whether the isochorismate pathway-related metabolic differences that we observed for *sid2*, *eds5,* and, particularly, *pbs3* (Figs. 4 and 7*A*), would result in an alteration of these immune characteristics or whether the mutants would behave similarly. Therefore, the manifestations of these characteristics were directly compared in the three mutants. First, attenuated NHP, Pip, and camalexin accumulation at 10 hpi was observed for both *sid2*, *eds5*, and *pbs3* (Figs. 7, *B* and *C*, and S6). Second, over-accumulation of NHP, NHPGE, and camalexin at later infection stages was observed in all of the mutants, with a somewhat smaller NHP over-accumulation in *pbs3* than in *sid2* or *eds5* (Figs. 7, *B* and *C*, and S6). Third, a strongly attenuated accumulation of NHPG following *Psm* inoculation was detectable in each of the mutants (Fig. 7*B*). Further, naïve (previously unstressed) *sid2*, *eds5*, and *pbs3* plants showed a very similar extent of compromised basal resistance toward bacterial challenge (Fig. 8*A*; water-treated plants). Moreover, treatments with exogenous NHP resulted in a similar degree of SAR towards *Psm* infection in the three mutant types, which was, however, significantly weaker than the SAR response in the wildtype (Fig. 8*A*). In addition, the biological induction of SAR in upper rosette leaves following an inducing inoculation of lower leaves with *Psm* was attenuated to similar degrees in the *sid2*, *eds5*, and *pbs3* mutants (Fig. 8*B*).

Together, these results indicate that the proposed second PBS3-independent branch of ICS1-dependent SA biosynthesis that leads to the accumulation of unconjugated SA at later infection stages barely affects plant basal immunity or SAR. Moreover, it is reasonable to assume that the described metabolic disturbances, concerning the biosynthesis of NHP and camalexin, and the glucosylation of NHP are caused by the most common feature of the three mutants - their failure to exert the predominant first (ICS1-, EDS5-, and PBS3-dependent) branch of isochorismate-derived SA biosynthesis.

## Discussion

**Figure 9:**
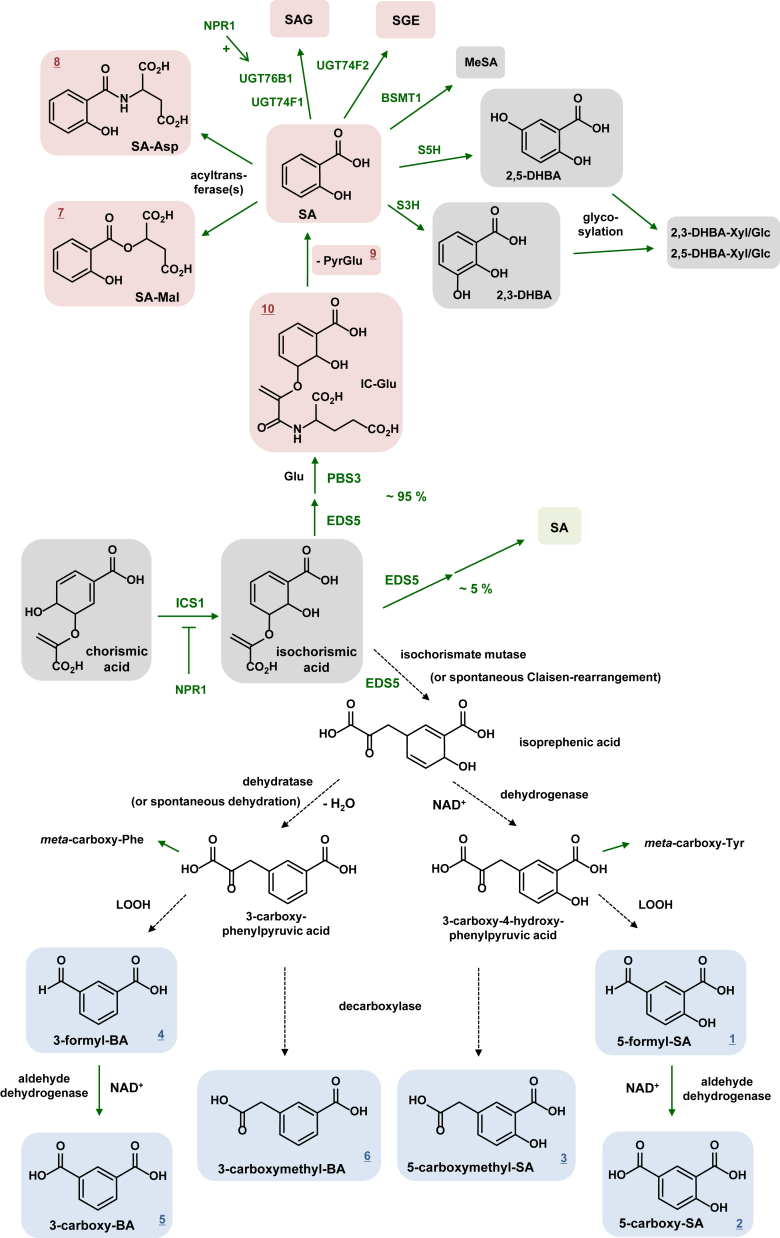
**Proposed scheme of biotic stress-inducible isochorismate synthase-associated metabolism in Arabidopsis.** The biotic stress-inducible ICS isoform ICS1 catalyzes chorismate to isochorismate conversion in the chloroplast. The MATE transporter EDS5 presumably exports isochorismate out of the chloroplast. Both ICS1 and EDS5 are necessary for the accumulation of ICS-pathway products. *Upper part*: The cytosolic acyl acid amido synthetase PBS3 mediates the major part (∼95%) of SA biosynthesis *via* IC-Glu formation, which eliminates Pyr-Glu to form SA. SA can be in turn converted to glucose derivatives (SAG, SGE), other conjugates (SA-Mal, SA-Asp), methyl salicylate, and hydroxylated to dihydroxybenzoic acids. In addition, a moderate PBS3-independent path (5%) of ICS1/EDS5-dependent SA biosynthesis (that primarily produces unconjugated SA; *green* background) exists. *Bottom part*: isochorismate can also re-arrange to isoprephenate, which might either undergo dehydration to form 3-carboxyphenylpyruvate or be oxidised to 3-carboxy-4-hydroxyphenylpyruvate. The latter two compounds can be transaminated to *meta*-carboxy-Phe and *meta*-carboxy-Tyr, respectively, and are also plausible precursors for the detected *meta*-substituted benzoic acid (3-FBA, 3-CBA, 3-CMBA) and salicylic acid derivatives (5-FSA, 5-CSA, 5-CMSA). The formation of the formyl-SA/BA derivatives from phenylpyruvate-intermediates might by mediated by membrane-derived lipid hydroperoxides (LOOH). Arabidopsis leaves possess oxidase activity to convert the formyl-into carboxy-SA/BA derivatives. Decarboxylase reactions could convert the phenylpyruvate-intermediates into the carboxymethyl-SA/BAs. The compounds highlighted with *red* (fail to accumulate in *pbs3* mutants) and *blue* (over-accumulate in *pbs3*, presumably due to redirection of metabolic flow) backgrounds have been analytically detected in this study. The accumulation of most of the ICS pathway products is negatively regulated by NPR1. Compounds highlighted in *grey* or compounds without background have not been analysed. The *green* arrows symbolize well-established conversions *in planta*, dashed arrows symbolize hypothetic conversion that are, nevertheless, plausible because of literature data. Please refer to the main text for further details.

The conversion of the key shikimate pathway intermediate chorismate to its isomer isochorismate is catalyzed by isochorismate synthase. Arabidopsis possesses two ICS isoforms, ICS1 and ICS2. While *ICS1* is stress-inducible and required for more than 99% of the inducible biosynthesis of SA in *P. syringae*-inoculated leaves (Fig. 7*A*), *ICS2* is constitutively expressed and does not contribute to stress-induced SA accumulation (5, 18, 19). Some bacterial ICS are bifunctional enzymes and possess, in addition to their chorismate-to-isochorismate-converting properties, an isochorismate pyruvate lyase (IPL) activity that directly produces SA from the originating isochorismate (20). However, both of the Arabidopsis ICSs were characterized as monofunctional isochorismate synthases that lack IPL activity and thus generate isochorismate as a reaction product ((4, 6); Fig. 9).

### Biosynthesis of biotic stress-inducible, *meta*-substituted formyl-, carboxy-, and carboxymethyl-benzoic acid and -salicylic acid derivatives in Arabidopsis

In the present study, we have conducted comparative metabolite analyses of wild-type plants and the *ICS1*-defective *sid2* mutant to study isochorismate-derived metabolism in Arabidopsis leaves. Based on mass spectrometric and IR spectroscopic identification, we report the occurrence of the three 5-substituted SA derivatives 5-FSA, 5-CSA, 5-CMSA, and the three analogous BA derivatives 3-FBA, 3-CBA, and 3-CMBA in Arabidopsis (Fig. 1). These newly identified plant metabolites, which carry formyl-, carboxy-, and methylcarboxy moieties at the *meta*-positions relative to the carboxy groups of the common SA or BA core units, accumulate in leaves upon inoculation with compatible and incompatible *P. syringae* strains and were collectively designated as *meta*-substituted SA- and BA-derivatives (Figure 1, Figure 4, Figure 5 and 9; Fig. S5). The compromised accumulation of these compounds in the *sid2* mutant indicates that the pathogen-inducible generation of isochorismate *via* ICS1 is necessary for their biosynthesis (Figs. 1*A*, and 4, *A* and *B*). Moreover, their formation requires the function of the chloroplast envelope-resident MATE transporter EDS5 (Fig. 4, *A* and *B*), which is presumed to mediate isochorismate transport out of plastids (21). Therefore, isochorismate exported from plastids might be required for the production of these substances. Likewise, the stress-inducible biosynthesis of SA strictly depends on both *ICS1* and *EDS5* (Fig. 7*A*). Biochemical and structural characteristics therefore appeared in line with a potential biosynthetic precursor function of SA for the formation of the six identified *meta*-substituted aromatics. However, exogenously administered SA or deuterium-labeled SA were not converted to any of the *meta*-substituted SA- and BA-derivatives in plants, irrespective of whether their leaves were inoculated with *P. syringae* or not (Fig. 3). Moreover, NahG overexpressing plants that, due to the SA hydroxylase activity introduced by the *NahG* transgene (46), rapidly degrade generated SA into catechol did not show compromised accumulation of the *meta*-substituted derivatives (Fig. 4, *A* and *B*). These findings indicate that SA does not serve as a biosynthetic precursor for the six *meta*-substituted aromatic acids (Fig. 9).

Relatively few *meta*-substituted aromatic natural products have been identified so far (51). Whereas the formation of 3-formyl-Tyr was described in the gamma-proteobacterium *Pseudoalteromonas tunicate* (52), *meta*-Tyr has been identified as an allelopathic constituent of root exudates of the grass species *Festuca rubrum* (53). Notably, the occurrence of four *meta*-substituted aromatic amino acids, *meta*-carboxy-Phe, *meta*-carboxy-Tyr, (3-carboxyphenyl)-glycine, and (3-carboxy-4-hydroxyphenyl)-glycine was reported in some plant species of the Brassicaceae, Resedaceae, Cucurbitaceae, and Iridaceae families (54). Incorporation of radiolabels into *meta*-carboxy-Phe and *meta*-carboxy-Tyr upon feeding with ^14^C- and ^3^H-labeled shikimate suggested that the *meta*-substituted aromatic amino acids are synthesized *via* the shikimate pathway (55). A closer analysis of the observed labeling patterns supported a biosynthesis from isochorismate and the thereof derived, putative intermediate isoprephenate. Subsequently produced 3-carboxyphenylpyruvate and 3-carboxy-4-hydroxyphenylpyruvate were proposed as specific biosynthetic precursors for the formation of *meta*-carboxy-Phe and *meta*-carboxy-Tyr, respectively (55). Consistently, Wu and colleagues (2024) recently provided biochemical evidence that the Arabidopsis aminotransferase REVERSAL OF SAV3 PHENOTYPE 1 (VAS1) catalyzes the transfer of amino groups from aromatic amino acids to the oxoacids 3-carboxyphenylpyruvate and 3-carboxy-4-hydroxyphenylpyruvate to generate *meta*-carboxy-Phe and *meta*-carboxy-Tyr, respectively (56). By employing an *ics1ics2* double mutant, this study further corroborated that the meta-carboxy aromatic amino acids are derived from isochorismate, and demonstrated that the VAS1-catalyzed reaction contributes to the homeostasis of aromatic amino acids (56).

Based on the present results and the above-outlined previous reports on isochorismate-derived metabolism, we propose a plausible biosynthetic scheme for the formation of the six *meta*-substituted SA/BA derivatives identified in our study (Fig. 9). Isochorismate that accumulates as a consequence of stress-inducible ICS1-activity might be converted to isoprephenate. This rearrangement is analogous to the well-documented chorismate mutase reaction that converts chorismate to prephenate (57, 58), and might be catalyzed by a putative isochorismate mutase. Interestingly, an enzymatic activity that converted isochorismate to isoprephenate has been previously identified in protein extracts of woodland tobacco (*Nicotiana sylvestris*) (59). On one hand, isoprephenate could be dehydrated to 3-carboxyphenylpyruvate by a dehydratase in analogy to the preserved prephenate dehydratase-catalyzed reaction involved in plant Phe biosynthesis (58, 60). 3-carboxyphenylpyruvate could then serve as the precursor for the biosynthesis of the three *meta*-substituted benzoic acid derivatives, 3-formyl-BA, 3-carboxy-BA, and 3-carboxymethyl-BA (Fig. 9). On the other hand, isoprephenate might be enzymatically oxidized to 3-carboxy-4-hydroxyphenylpyruvate *via* a dehydrogenase-catalyzed reaction in which aromatization occurs by retention of the phenolic hydroxyl group. A comparable aromatization reaction represents the conversion of arogenate to Tyr catalyzed by arogenate dehydrogenase (61). 3-carboxy-4-hydroxyphenyl-pyruvate represents a plausible precursor for the biosynthesis of the three *meta*-substituted salicylic acid derivatives 5-formyl-SA, 5-carboxy-SA, and 5-carboxymethyl-SA (Fig. 9).

Chemically, the conversion of isochorismate to isoprephenate constitutes a Claisen-type of [3,3]-sigmatropic rearrangement with an energetically favored transition state (51). Consistently, a conversion of isochorismate to 3-carboxyphenylpyruvate, presumably *via* isoprephenate as the intermediate, has been observed to proceed non-enzymatically at temperatures of 100 °C as a main route of thermal isochorismate degradation (62, 63). Whether a spontaneous conversion of isochorismate to carboxyphenylpyruvate occurs under milder biological conditions is unclear, but a spontaneous, non-enzymatic formation of carboxyphenylpyruvate from isochorismate cannot be ruled out. However, because of the necessity of an electron acceptor in the proposed oxidation step of isoprephenate, the formation of 3-carboxy-4-hydroxyphenylpyruvate is unlikely to occur in a non-enzymatic manner (Fig. 9).

We propose that the two putative intermediates 3-carboxyphenylpyruvate and 3-carboxy-4-hydroxyphenylpyruvate are further converted to the identified *meta*-substituted BA- and SA-derivatives, respectively. As phenylpyruvate derivatives, 3-carboxyphenylpyruvate and 3-carboxy-4-hydroxyphenylpyruvate are convertible to the phenylacetate variants, 3-CMBA and 5-CMSA, respectively, *via* oxidative decarboxylation reactions (Fig. 9). Notably, an analogous biochemical conversion of phenylpyruvate to phenylacetate takes place in yeast as part of the Ehrlich pathway, a reaction sequence that mediates Phe catabolism. Herein, phenylpyruvate is decarboxylated by phenylpyruvate decarboxylase to phenylacetaldehyde which is in turn oxidized to phenylacetate by an aldehyde dehydrogenase (64, 65). A similar two-step conversion of phenylpyruvate to phenylacetate was identified in the soil bacterium *Achromobacter eurydice* (66).

The peroxidation of membrane lipids is a common metabolic process in plant tissue suffering from biotic or abiotic stress (67). In the course of the interaction of Arabidopsis leaves with compatible or hypersensitive response-inducing *P. syringae* strains, lipid hydroperoxides (LOOH) and other lipid peroxidation products are generated non-enzymatically following the production of reactive oxygen species (68, 69). Interestingly, chemical conversion of phenylpyruvate to benzaldehyde and phenylacetate was observed in the presence of LOOH derived from linolenic acid (70). Analogously, it is conceivable that lipid peroxidation products mediate an oxidative cleavage of 3-carboxyphenylpyruvate and 3-carboxy-4-hydroxyphenylpyruvate to the benzaldehydes 3-FBA and 5-FSA, respectively (Fig. 9). Our feeding experiments show that exogenously added 3-FBA and 5-FSA are rapidly oxidized to the respective carboxy-derivatives 3-CBA and 5-CSA in the leaf tissue, strongly suggesting that an aldehyde dehydrogenase activity is present in Arabidopsis that mediates these conversions (Fig. 3*E*). Because non-inoculated, uninduced plants were employed in this feeding assay, this presumed aldehyde dehydrogenase might be constitutively expressed in leaves. Interestingly, compared to the wildtype, the SA-deficient NahG plants over-accumulate the formyl variants 3-FBA and 5-FSA in response to *P. syringae* inoculation, while they accumulate reduced amounts of the carboxy-derivatives 3-CBA and 5-CSA (Fig. 4, *A* and *B*). This might indicate that the activity or expression of the proposed aldehyde dehydrogenase is modulated by SA.

Thus, a combination of enzymatic and non-enzymatical conversion steps might be involved in the synthesis of the six identified *meta*-substituted SA/BA-derivatives from isochorismate (Fig. 9). Theoretically, it is possible that during the infection process, bacterial enzymes catalyze specific steps of the proposed pathway in combination with plant enzymes. Such a scenario is supposed to imply a positive correlation between bacterial numbers in leaves and the pathway products. In particular, our finding that incompatible bacterial inoculation, which is associated with a limited bacterial propagation due to efficient activation of plant defense (71), induces the accumulation of *meta*-substituted SA/BA-derivatives to a similar extent than compatible bacterial infection argues against this possibility. The earlier production of meta-substituted SA/BA-derivatives in the incompatible *Psm avrRpm1*-Col-0 interaction compared to the compatible *Psm*-Col-0 interaction is reminiscent of the accumulation patterns of different plant-derived metabolites, such as indolics or tocopherols (69, 72), and rather suggests that meta-SA/BAs are generated by an active plant response that is elicited following pathogen inoculation (Fig. S5*A*).

Under pathogen-inoculation conditions, a major portion of the ICS1-generated isochorismate is used for the PBS3-mediated biosynthesis of SA and the SA-derived derivatives such as SAG, SGE, SA-Mal, and SA-Asp (Fig. 5). For example, out of the total levels of measured SA derivatives, about 2% SA, 90% SAG, 3% SGE, 2% of SA-Mal, and 1% SA-Asp accumulated in the Col-0 wildtype at 48 h post *Psm* inoculation, while the contribution of the *meta*-substituted SA/BA-derivatives together was less than 2% (Fig. 5). In the *pbs3* mutant, however, the metabolic route towards SA is largely blocked (Figs. 4*C*, and 7*A*). A redirection of the isochorismate-derived metabolic flow toward the production of the *meta*-substituted SA/BA-derivatives would explain our findings that the latter compounds strongly over-accumulate in *P. syringae*-inoculated *pbs3* mutant plants (Fig. 4, *A* and *B*). Our model also proposes 3-carboxyphenylpyruvate and 3-carboxy-4-hydroxyphenylpyruvate as central pathway intermediates that are derived from isochorismate (Fig. 9). This would explain why a chemically identical set of BA and SA derivatives carrying formyl-, carboxy-, and carboxymethyl groups at the *meta*-positions of their aromatic rings are generated in a strikingly concerted mechanistic and temporal manner in response to pathogen attack (Figs. 4, *A* and *B*, and 5). Whether the two previously described 3-carboxyphenylpyruvate- and 3-carboxy-4-hydroxyphenyl-pyruvate-derived metabolites *meta*-carboxy-Phe and *meta*-carboxy-Tyr (56), respectively, accumulate upon biotic stress remains to be determined. A previous microarray study indicates a modest increase of transcripts of the *VAS1* gene in *Psm*-infected Col-0 leaves (Fig. S8; (73)).

### SA-Mal and SA-Asp are biosynthesized from SA and require PBS3 for their formation

Another hitherto undescribed SA derivative that we found to occur in Arabidopsis leaves is the malate ester of SA, salicyloyl-malate (SA-Mal) (Fig. 2, *A*–*D*). Just as the amide salicyloyl-aspartate (SA-Asp) that was previously described as a plant natural product (37, 38, 39, 40, 41), SA-Mal accumulated upon *P. syringae* inoculation in strict dependency of the SA biosynthetic genes *ICS1*, *EDS5*, and *PBS3* (Fig. 4*C*). Moreover, the SA-degrading NahG plants were unable to accumulate both SA-Mal and SA-Asp (Fig. 4*C*). In addition, exogenously administered (deuterium-labeled) SA was readily converted to the (deuterium-labeled) SA-Mal and SA-Asp variants in *P. syringae* inoculation leaves (Figs. 2, *B* and *E*, and 3). These findings demonstrate that both SA-Mal and SA-Asp are synthesized from SA as a direct or indirect biosynthetic precursor.

A prominent malate ester generated in the vegetative tissue of Arabidopsis is the UV-B protective metabolite sinapoyl-malate, which is synthesized from the glucose ester sinapoyl-1-glucose by a serine carboxypeptidase-like (SCPL) acyltransferase (74, 75). Whether SA-Mal is synthesized by similar biochemical principles remains to be determined. Parallel *P. syringae*-induced accumulation patterns of SGE and SA-Mal, though, do not argue against the possibility that SGE could function as an SA-Mal precursor ((11); Figs. 4, and S2).

The acyl acid amido synthetase GH3.5 was previously implicated in the formation of SA-Asp. GH3 enzymes generally catalyze the ATP-dependent adenylation of the carboxylic group of organic acid and a subsequent displacement of AMP from this adenylated intermediate by the amine group of an *L*-amino acid, so that an amide conjugate is formed as a product (76). A role for GH3.5 in the biosynthesis of SA-Asp was supported by *in vitro* assays, since the purified GH3.5 protein showed adenylation activity towards SA, indole acetic acid (IAA), and several other acid substrates, and furthermore catalyzed the formation of SA-Asp from SA and Asp (40, 41, 42). Also, over-expression of *GH3.5* in Arabidopsis resulted in increased accumulation of SA-Asp. However, since *gh3.5* knockout plants did not exhibit reduced SA-Asp levels (40), the biosynthesis of SA-Asp in Arabidopsis seems not yet fully understood. Nevertheless, our analyses illustrate that the pathogen-inducible generation of SA-Asp proceeds *via* isochorismate pathway-derived SA formation and *via* similar kinetical and regulatory principles than the generation of SA-Mal (Figs. 4*C*, 5*C*, and 9; Figs S2 and S4).

### Exogenous application of 5-carboxy-SA and SA-Mal has a moderate impact on plant immunity

To test possible immune-stimulating activities of the here-identified SA-related compounds, we applied commercially available (5-FSA, 5-CSA, 3-FBA, 3-CBA, 3-CMBA) or chemically synthesized (SA-Mal) substances in the same concentrations than SA (0.5 mM) to Arabidopsis plants and assessed the induction *PR1* gene expression and pathogen resistance to *P. syringae* (Fig. 6). Our results indicate that none of the compounds exhibits immune-inducing properties to a similar magnitude than SA. Out of the applied *meta*-substituted SA/BA derivatives, only 5-CSA exhibited a modest resistance-enhancing activity in wild-type plants, while 5-FSA, 5-CSA, 3-FBA, and 3-CBA, caused marginal effects on resistance- and/or *PR1*-induction in *sid2* plants. These results, together with our findings that they accumulate to low absolute levels and relatively late after pathogen inoculation (Fig. 5), suggest a minor role for *meta*-substituted SA/BA derivatives in inducible plant immunity.

In accordance with earlier results (30), we found that the 3-substituted SA derivative 2,3-DHBA exerts moderate resistance- and *PR1*-inducing activities that were, however, markedly lower than the respective activities of SA. By contrast, the 5-substituted isomer 2,5-DHBA (gentisic acid), did not exert any immune-enhancing effect (Fig. 6, *B* and *C*). The weak or non-existent immune-activating properties of 5-FSA, 5-CSA, and 2,5-DHBA illustrate that a 5-substitution in the aromatic ring of SA can severely compromise the biological activity of the SA molecule. However, this is not necessarily true for every 5-substitution, because exogenous application of the synthetic SA derivative 5-chloro-SA resulted in marked elevation of *PR1* transcript levels and resistance-induction in plants (77). Consistently, *in vitro* binding assays showed that the SA receptor NPR1, which mediates SA-triggered immune responses (78), tightly interacts with 5-chloro-SA in addition to SA (15). By contrast, benzoic acids that lack the 2-hydroxy group at the *ortho*-position of the benzene ring do not bind to NPR1 and hardly exhibit immune-stimulating activity (15, 77). The 2-hydroxy group as a key structural prerequisite for the immune-activating properties of salicylates is absent in 3-FBA, 3-CBA, and 3-CMBA, which offers an explanation for the observation that the three exogenously applied *meta*-substituted BAs had virtually no impact on immune responses (Fig. 6, *A* and *C*).

We assessed a marked resistance enhancement in plants treated with 0.5 mM SA-Mal, but the resistance effect was lower than for SA treatments (Fig. 6*E*). When infiltrated into leaves, a substantial portion of the applied SA-Mal was relatively rapidly hydrolyzed to SA, which might indicate the presence of a respective esterase activity in the plant tissue (Fig. 6*G*). Therefore, we cannot exclude that the observed resistance effects observed in response to exogenously applied SA-Mal solutions were due to the release of free, signal-active SA. Modest resistance-activation effects were also reported when SA-Asp was fed exogenously to plants (37, 40). However, prerequisites for convincing conclusions about the biological functions of SA-Mal and SA-Asp are the elucidation of their biosynthesis and the functional characterization of appropriate gene mutant lines that specifically lack the accumulation of these SA conjugates.

### A moderate PBS3-independent path to isochorismate-derived SA operates in Arabidopsis leaves at later stages of bacterial infection

Bacterial IPLs eliminate the enolpyruvyl side chain of their substrate isochorismate to generate SA and pyruvate, but attempts to identify plant enzymes with IPL activities were unsuccessful in the past (20, 79). In 2019, two parallel studies reported on a plant-specific circuit strategy for isochorismate to SA conversion *via* PBS3-catalysed conjugation of *L*-Glu to isochorismate. SA is then synthesized from the resulting intermediate IC-Glu by elimination of Pyr-Glu (21, 22). In combination with detailed *in vitro* biochemical characterization of PBS3, both studies primarily relied on the phenotypic and metabolic characterization of different Arabidopsis autoimmune mutant lines that over-accumulated, in a *PBS3*-dependent manner, IC-Glu, Pyr-Glu, and SA in the absence of biotic stress (21, 22). The significance of *PBS3* for basal and effector-triggered immunity to *P. syringae* was established in preceding studies, which also found that accumulation of SA and SAG, and *PR1* gene expression following microbial inoculation was significantly reduced in *pbs3* mutants (45, 80, 81). Our study confirms the importance of PBS3-mediated SA generation and the previously reported biochemistry *via* IC-Glu formation and subsequent release of Pyr-Glu in a well-defined plant-pathogen interaction context. Our time-course analysis shows that rises of IC-Glu and Pyr-Glu are detectable at 10 h post *Psm* inoculation and then become gradually larger until 48 hpi (Fig. 5*C*).

A direct comparison of the levels of totally accumulating SA (*i.e.*, the sum of SA and SA conjugates) showed that the increases of total SA in *sid2*, *eds5*, and *pbs3* following *Psm* inoculation amounted to ∼0.5%, ∼1.5% and ∼5.5% of the corresponding increases in the wildtype Col-0 at 48 hpi (Fig. 7*A*). This suggests that almost the whole pathogen-induced biosynthesis of SA proceeds *via* ICS1 and, almost as stringently, *via* EDS5. The residual, faint amounts of SA accumulating in *sid2* leaves, which are below the basal levels of the wildtype (Fig. 7*A*), might be generated by the activity of the second Arabidopsis isochorismate synthase isoform, ICS2, or *via* the PAL pathway (5, 17). Nevertheless, these data illustrate that blockage of the isochorismate pathway in the leaves of the *sid2* and *eds5* mutants does not lead to a significant compensation of SA biosynthesis *via* alternative routes (Fig. 7*A*), such as the PAL pathway. Moreover, increased PAL activity upon pathogen detection has been functionally associated with early cell wall-based defenses and lignification events (82).

Notably, ∼5% of this isochorismate-derived SA accumulates in a PBS3-independent manner (Fig. 9). Just as *eds5*, *pbs3* mutants are strongly compromised in the accumulation of the SA conjugates SAG, SGE, SA-Mal and SA-Asp. However, *pbs3* only shows compromised accumulation of unconjugated SA at earlier infection phases (6 and 10 hpi), but accumulates free SA to wildtype-like levels (24 hpi) or to even higher amounts (48 hpi) at later interaction periods (Figs. 4*C*, and 7*A*). This suggests the existence of a moderate PBS3-independent branch to SA that operates later in the plant-pathogen interaction phase and mainly produces unconjugated SA. Our data are consistent with previous studies that, depending on the chosen pathogen strain and sampling time, either detected attenuated and not entirely compromised *P. syringae*-induced accumulation of free SA, or over-accumulation of free SA in *pbs3* mutants. In all of the cases, however, the accumulation of SAG was strongly impaired (45, 80, 81). The PBS3-independent accumulation of free SA might be explained by a non-enzymatic [1,5]-sigmatropic rearrangement of isochorismate to SA and pyruvate rather than by a PAL-associated replenishment pathway for SA (see argumentation above; (21, 51, 62)). A spontaneous chemical conversion of isochorismate to SA is expected to proceed by a lower reaction rate than an enzyme-assisted conversion *via* PBS3, and therefore could gain significance in later periods after a pathogen-induced ICS1 activation and a resulting isochorismate formation. This would explain the observed time-dependent accumulation characteristics of free SA in *pbs3* (Fig. 7*A*). The apparently compromised SA conjugation in *pbs3* might then be related to spatial or temporal inaccessibility of the spontaneously generated SA to conjugating enzymes.

Our data indicate that *sid2*, *eds5*, and *pbs3* plants share several immune-related features and metabolic characteristics that were previously described for the *sid2* mutants (10, 12, 18, 19, 49, 50). All of the mutants show a comparable attenuation of basal immunity to *P. syringae.* In addition, SAR induced biologically in plants *via* pathogen inoculation or chemically *via* exogenous treatment with the natural SAR hormone NHP is compromised to similar degrees in all of the mutant types (Fig. 8). With respect to the metabolic irregularities, the three mutant lines show reduced accumulation of the defense metabolites NHP, Pip and camalexin during the early *Psm*-Arabidopsis interaction phase (10 hpi), over-accumulation of NHP and its glucose ester NHPGE at later periods (from 24 hpi onward), and attenuated accumulation of the NHP glucoside NHPG throughout the studied plant-pathogen interaction (Figs. 7, *B* and *C*, and S6). These findings suggest that the defects in the predominant PBS3-dependent branch of SA biosynthesis are causative for the above-described immune defects and metabolic disturbances, while the PBS3-independent branch that results in a later accumulation of free SA does not markedly affect immune responses. This is consistent with previous conclusions that SA primes NHP and camalexin biosynthesis, particularly during earlier plant-pathogen interaction phases, and prevents an excess accumulation of NHP during later infection stages by promoting NHP to NHPG conversion (11, 12, 26, 50). In addition, the enhanced susceptibility phenotype of the immune-compromised lines *sid2*, *eds5*, *pbs3*, *npr1* or *NahG* might contribute to some of the observed over-accumulation patterns of metabolites at later time points (especially 48 hpi), because higher bacterial numbers in the tissue of susceptible plants likely exert enhanced response stimulation than in the tissue of the more resistant wild-type (Figs. 4 and 7). Nevertheless, as discussed here, regulatory principles and the re-direction of plant metabolic pathways in biosynthetic mutants exert strong influences on the production of metabolites in the plant lines under investigation.

### Metabolic accumulation patterns argue against a previously proposed involvement of EDS5 in the biosynthesis of NHP and associated transport processes

The biosynthesis of the SAR-inducing defense metabolite NHP proceeds in three enzymatic steps out of *L*-Lys (9). The first two steps convert *L*-Lys to Pip *via* the aminotransferase ALD1 and the reductase SARD4 (44, 83, 84). The subcellular localization of ALD1-and SARD4-fusion proteins thereby suggests a plastidial biosynthesis of Pip (85, 86). In a third step, the flavin-dependent monooxygenase FMO1 catalyses the *N*-oxidation of Pip to NHP (10), but the subcellular localization of FMO1 is unclear yet. However, considering the common occurrence of cytosolic or endoplasmic reticulum-located monooxygenation reactions (87), a transport of Pip out of the plastid across the plastid envelope might be necessary for the completion of NHP biosynthesis. A preliminary study by Rekhter and colleagues, which had been posted several years ago as a pre-print to bioRxiv but, to our knowledge, not been published so far in a peer-reviewed journal, proposed a model in which the chloroplast envelope-resident transporter EDS5 was involved in the export of Pip out of the plastid and in NHP biosynthesis. This rather speculative assumption was primarily based on reduced NHPG levels detected in *eds5* mutants upon UV light exposure (88), but some reviews already integrated this proposition into NHP biosynthesis-related models (*e.g.*, (89)). However, the strong accumulation of NHP in *Psm*-inoculated *eds5* mutants clearly exclude that EDS5 is necessary for NHP biosynthesis or involved in a possibly related transport of Pip out of plastids (Fig. 7*B*). The uniform accumulation characteristics of NHP and NHPG in *P. syringae*-inoculated *sid2*, *eds5*, and *pbs3* plants outlined above rather indicate that SA functions in the promotion of the early biosynthesis of NHP and the containment of NHP accumulation at later stages by supporting NHP to NHPG conversion (Fig. 7*B*).

### The different branches of the isochorismate pathway are negatively regulated by NPR1

Another striking metabolic feature we observed in this study was the over-accumulation of every of the ten characterized ICS1-dependent Arabidopsis metabolites (1–10) in the *npr1* mutant after *Psm* inoculation (Figs. 4, and S2). Likewise, SA and SGE over-accumulated in *npr1* in response to bacterial infection, whereas SAG did not (Fig. S2; (11)). In addition, the basal levels of 2,5-DHBA were found to be decreased in the *npr1* mutant (90). This illustrates that the SA receptor NPR1 has a profound impact on the Arabidopsis isochorismate pathway. The concerted negative impact of functional NPR1 on the *meta*-substituted SA- and BA-derivatives, SA, SA conjugates (SGE, SA-Mal, SA-Asp), and SA biosynthetic intermediates (IC-Glu, Pyr-Glu) indicates that NPR1 negatively regulates the isochorismate pathway at an early step upstream of isochorismate production, which could lead to an over-accumulation of isochorismate in *npr1* mutants, and consequently, enhanced production of isochorismate-derived compounds under pathogen infection conditions (Fig. 9). This hypothesis is consistent with the over-production of isochorismate in a *snc2npr1* double mutant that exhibits *snc2*-triggered activation of autoimmune pathways (21), and with the observed negative impact of functional *NPR1* on *ICS1* expression (90). Additionally, *NPR1* positively affects the strongly pathogen-inducible expression of *UGT76B1*, which encodes a glucosyltransferase that simultaneously glucosylates SA and NHP to their glucosides SAG and NHPG under biotic stress conditions (11, 26, 90). Quantitatively, SAG is the main accumulating SA derivative in Arabidopsis (Fig. 5; (9)), and an attenuated *UGT76B1* expression in *npr1* might thus redirect the SA metabolic pathway towards accumulation of free SA and its conjugates SGE, SA-Mal, and SA-Asp (Fig. 9). Similarly, the expression of the SA-5-hydroxylase gene *S5H* was promoted by *NPR1* (90), which might also contribute to the over-accumulation of SA and SA conjugates in *npr1* mutant plants due to metabolic backlog. Together, our study suggests an important function of NPR1 in the homeostasis of a whole set of differently regulated isochorismate pathway-derived metabolites in pathogen-attacked plants.

## Experimental procedures

### Seed material and plant cultivation

Arabidopsis (*A. thaliana*) plants were cultivated individually in pots containing a mixture of soil (Substrat BP3; Klasmann-Deilmann), vermiculite, and sand (8:1:1) in a growth chamber with a 10-h-day (9 _AM_ to 7 _PM_; 100 μmol m^−2^ s^−1^ photon flux density)/14-h-night cycle and a relative humidity of 60%. Day and night temperatures were 21 °C and 18 °C, respectively (10). The following Arabidopsis gene knock-out lines were employed in this study: *sid2* (*sid2-1*; (18)), *eds5* (*eds5-1*) (91), *pbs3* (*pbs3-2*; Salk_018225; (45))*, npr1* [*npr1-3*; Nottingham Arabidopsis Stock Centre (NASC) ID: N3802]. Moreover, the *NahG* overexpressing line (NahG) was used (46). All lines are in the Col-0 (N1092) background.

### Bacterial inoculation and treatment assays for the determination of disease resistance, metabolite analyses, and defence gene expression

*P. syringae* pv. *maculicola* strain ES4326 (*Psm*), *Psm* expressing the luxCDABE operon from *Photorhabdus luminescens* (*Psm lux*), and *Psm* expressing the *AvrRpm1* avirulence gene (*Psm avrRpm1*) were used for bacterial inoculation assays associated with metabolite analyses and bacterial growth assessments, respectively. The bacterial strains were cultivated at 28 °C in King’s B medium with the appropriate antibiotics as previously described (10, 34, 92, 93). For plant inoculation, bacterial suspensions from overnight cultures were washed and diluted with 10 mM MgCl_2_ to final optical densities at 600 nm (OD_600_) of 0.005 (*Psm* and *Psm AvrRpm1*) and 0.001 (*Psm* *lux*). Bacterial suspensions were infiltrated into the abaxial sides of Arabidopsis rosette leaves with needleless syringes between 10 _AM_ and 11 _AM_.

For the determination of metabolite contents in Arabidopsis leaves following bacterial attack, suspensions of *Psm* (OD_600_ = 0.005) were infiltrated into three rosette leaves of 5-week-old plants. As a mock-control treatment, a 10 mM MgCl_2_ solution was infiltrated instead. The treated leaves were harvested at different times (6, 10, 24 or 48 h post treatment), leaf fresh weights (FW) were determined, and harvested leaves shock-frozen in liquid nitrogen. Each replicate sample consisted of six leaves from two different plants. Four to five replicate samples were usually analyzed in each experiment.

For assessments of resistance to bacterial infection, purified suspensions of the bioluminescent *Psm* *lux* (OD_600_ = 0.001) were syringe-infiltrated into three Arabidopsis rosette leaves (87, 88). Bacterial numbers were determined 2.5 days post-inoculation by measuring the luminescence of leaf discs punched out of inoculated leaves (one disc per inoculated leaf) with a Sirius FB12 luminometer (Berthold Detection Systems, http://www.titertek-berthold.com). As a measure of disease susceptibility, bacterial numbers were assessed as relative light units (rlu) per cm^2^ leaf area. At least 15 replicate leaf samples were assayed for each genotype.

To assess the induction of resistance and defense gene expression following treatments with SA (Sigma, S5922), *meta*-substituted SA/BA derivatives [3-FBA (Acros, 296560010), 3-CBA (Alfa Aesar, A14445), 3-CMBA (Fluorochem, 446214), 5-FSA (Sigma, F17601), 5-CSA (Sigma, 797057)], other SA derivatives [2,3-DHBA (Acros, A0350739), 2,5-DHBA (Fluka, 537960), SA-Mal (chemically synthesized, see below)], malate (Roth, 3034.1), and fumarate (Fluka, 47900), 0.5 M stock solutions in ethanol were freshly prepared, and diluted with water to a working concentration of 0.5 mM. The 0.5 mM solutions were then infiltrated into three rosette leaves of a given Arabidopsis plant. As a mock-control treatment, an aqueous solution containing 0.1% ethanol was infiltrated. For the assessment of defence gene expression, six leaves from two plants were harvested 4 h after the treatment to obtain one replicate sample. To assess resistance induction, the treated leaves were inoculated with *Psm* *lux* 4 h after the pre-treatment, and bacterial growth determined *via* bacterial luminescence as described above.

To examine SAR establishment following exogenous *N*-hydroxypipecolic acid application, 10 ml of a freshly prepared 1 mM NHP solution or 10 ml of water (control treatment) was pipetted onto the soil of the individually cultivated plants (10). Challenge inoculations with *Psm* *lux* were performed 1 day later as described above. To assess SAR following a bacterial inducer inoculation, three lower rosette leaves of a given plant were inoculated with *Psm* (OD_600_ = 0.005) or mock-infiltrated with 10 mM MgCl_2_. Two days later, three upper leaves were challenge-inoculated with *Psm* *lux* (OD_600_ = 0.001), and the numbers of *Psm* *lux* assessed 2.5 days later as described above (93).

### Chemical synthesis of salicyloyl-malate (SA-Mal)

SA-Mal (racemic O-salicyloyl malic acid) was obtained by a three-step chemical synthesis from malic acid and O-benzyl salicylic acid. The synthesis and spectroscopic characterization of SA-Mal are outlined in detail in Fig. S3.

### Metabolite analysis by GC-MS or GC-FTIR *via* vapor phase extraction and analyte derivatization by methylation

The initial comparative metabolite analyses and the quantitative determination of the levels of the thereby identified *meta*-substituted SA/BA derivatives (5-FSA, 5-CSA, 5-CMSA, 3-BSA, 3-BSA, 3-CMBA), SA-Mal, SA-Asp, Pyr-Glu, and IC-Glu were performed *via* a vapor-phase extraction-based work up of leaf extracts according to Schmelz and colleagues (43), coupled with subsequent GC-MS or GC-FTIR analysis of the resulting derivatized samples (10, 44). 150 to 200 mg of shock-frozen leaf material (pooled from six leaves) was ground to a fine powder with a pre-chilled ball mill and immediately extracted with 600 μl of H_2_O:1-propanol:HCl (1:2:0.005; v/v/v) that was pre-heated to 70 °C and contained 100 ng of deuterium-labeled SA (D_6_-SA; Sigma-Aldrich, S16796) and dihydrojasmonic acid (TCI, D3225) as internal standards for the routine procedure [The D_6_-SA standard was, however, omitted from the extraction buffer when *in planta* labeling experiments with deuterated SA were analyzed. Please also note that D_6_-SA rapidly exchanges the two acidic deuterium atoms by hydrogens in protic solvents and thus in principle prevails as D_4_-SA in solution (Fig. S4).] After vortexing for 15 s, 1 ml methylene chloride was added, the suspension vigorously mixed by further vortexing for 30 s, and centrifuged at 14,000*g* for 1 min to facilitate phase separation. The lower organic phase was removed, dried over ∼10 mg of Na_2_SO_4_, and incubated for 5 min at room temperature with 4 μl of 2 M trimethylsilyl-diazomethane in hexane (Sigma-Aldrich), which converts analytes containing carboxylic acid groups into the corresponding methyl esters. The methylation reaction was stopped by the addition of an excess of acetic acid (4 μl of a 2 M solution in hexane), and the sample was subsequently subjected to a vapor phase extraction procedure using a volatile collector trap packed with Porapak-Q absorbent (VCT-1/4X3-POR-Q; Analytical Research Systems) (43). For that purpose, the sample was heated to 70 °C under a steady stream of nitrogen until complete evaporation of the solvent. The temperature was then increased to 200 °C for 2 min. The absorbed metabolites were eluted from the collector column with 1 ml methylene chloride. The collection was concentrated to 30 μl under a nitrogen stream and transferred to GC-vials. For gas chromatographic separation, 4 μl of the sample was injected into a 7890A GC (Agilent Technologies) equipped with a ZB5 MS capillary column (Zebron). GC injector temperature was set to 250 °C, a constant flow of helium (1.2 ml/min) was applied and the following temperature program was used: 50 °C/3 min with 8 °C/min to 240 °C, with 20 °C/min to 320 °C/3 min.

Mass spectra were recorded with a 5975C mass spectrometric detector (Agilent Technologies) in the electron ionization mode at 70 eV. The GC-MS data was evaluated using MSD ChemStation software version E.02.01.1177 (Agilent Technologies). For quantitative analysis, peaks of analytes and internal standards from selected ion chromatograms were integrated: 5-FSA (*m/z* 148), 5-CSA (*m/z* 178), 5-CMSA (*m/z* 133), 3-FBA (*m/z* 133), 3-CBA (*m/z* 163), 3-CMBA (*m/z* 177), SA-Mal (*m/z* 120), SA-Asp (*m/z* 121), Pyr-Glu (*m/z* 142), IC-Glu (*m/z* 142), D_4_-SA (internal standard; *m/z* 124), dihydrojasmonic acid (internal standard; *m/z* 156). Experimentally determined correction factors reflecting the ratios of areas of internal standard to analyte were considered for the quantification of 5-FSA, 5-CSA, 3-FBA, 3-CBA, 3-CMBA, and SA-Mal. Due to the unavailability of authentic compounds for 5-CMSA, SA-Asp, Pyr-Glu, and IC-Glu, correction factors were estimated considering the proportion of the selected *m/z*-value on the total ions of the mass spectra. All calculated values were related to the fresh weight (FW) of the leaf samples.

GC-FTIR spectra were acquired using a Hewlett-Packard 6890 Series GC coupled with an IRD3 infrared detector manufactured by ASAP Analytical (Analytical Solutions and Providers), as detailed previously (44). The GC-specific settings and the column were the same as described above, except that the flow rate of the helium carrier gas was 2 ml min^−1^. Infrared spectra were recorded from 4000 to 600 cm^−1^ with a resolution of 16 cm^−1^ and a scan rate of 8 scans per second. The IRD method parameters were as follows: Resolution = 16; Apodisation = Triangle; Phase correction = Mertz; Zero-Fill = 1; Co-Add = 2. The temperature of the transfer line and flow cell were both set to 250 °C, and nitrogen was used as sweep gas. The FTIR data were analyzed with the software Essential FTIR© (v3.10.037; Operant LLC).

### Metabolite analysis by GC-MS *via* analyte derivatization by trimethylsilylation

The quantitative assessment of the leaf levels of SA, SAG, SGE, NHP, NHPG, NHPGE, and camalexin was performed by a GC-MS-based analysis of trimethylsilylated analytes as described in detail in previous studies (10, 11). The absolute metabolite levels were related to the leaf FW.

### Metabolite analysis by LC-q-TOF-MS

The harvested, shock-frozen leaves were grounded to a fine powder with a ball-mill in a 2 ml Eppendorf tube. 600 μl of MeOH:H_2_O (80:20, v/v) was added, the sample was thoroughly vortexted and extracted for a further 10 min on a rotatory shaker at 4 °C. After centrifugation at 14000 rpm, the supernatant was carefully removed and the pellet extracted again with 600 μl of MeOH:H_2_O (80:20, v/v). The solvent of the combined extracts was evaporated at 30 °C under vacuum using a ScanSpeed vacuum centrifuge (Labogene ApS). The dry residue was redissolved in 100 μl of LC-MS-grade MeOH:H_2_O (80:20, v/v) and the solution filtered through a nylon centrifugal filter (VWR, 516-0233) at 14000 rpm. 5 μl of the filtrate was injected into a 1260 Infinity II Prime LC coupled to a 6540-quadrupole time-of-flight mass spectrometer, which was equipped with a dual electrospray ionization (ESI) source (Agilent Technologies). For chromatographic separation, an InfinityLab Poroshell 120 EC-C18 column (3.0 × 100 mm i.d., 2.7 μm particle size) was used. The mobile phase solvents were HPLC-grade water (solvent A) and acetonitrile (solvent B), both supplemented with formic acid (0.1% v/v). A gradient program was applied for the separation of the analytes, in which the percentage of solvent B was linearly changed as follows: 0 min, 5%; 1 min, 5%; 6 min, 25%; 12 min, 25%; 16 min, 50%; 20 min, 75%; 23 min, 95%; 26 min, 95%; 28 min, 5%; 34 min, 5%. The flow rate and column temperature were set to 0.7 ml min^−1^ and 40 °C, respectively. The MS analysis was performed in the negative ionization mode. Nitrogen was used as the nebulizer and drying gas and was set to 40 psi and 10 l/min, respectively. The drying gas temperature was set to 325 °C. The instrument ion optic voltages were as follows: fragmentor 130 V, skimmer 65 V and octopole RF 750 V. The capillary and nozzle voltages were 3500 V and 300 V, respectively. The data was acquired in the MS^1^ mode between *m/z* 50 and *m/z* 1700 and processed using MassHunter software (Agilent). For relative quantification, the peak areas of specific extracted ion chromatograms ([M-H]^−^ ions or other main fragment ions of the analytes were integrated and related to the sample FW (Fig. S2).

### Determination of PR1 transcript levels by RT-qPCR analysis

Leaf transcript levels of *PR1* were determined by RT-qPCR analysis using 50 mg of frozen and ground leaf tissue. The RNA isolation, cDNA synthesis, and RT-qPCR steps have been previously outlined in detail (49, 83). The *POLYPYRIMIDINE TRACT-BINDING PROTEIN 1* (*PTB1*) gene was used as a reference gene. Fig. S7 lists the primers used for RT-qPCR analysis. Two technical replicates were assessed for one sample, and their means were taken to obtain expression values for one biological replicate. The *PR1* transcript levels were expressed relative to the mean value of the Col-0-mock-control sample (Fig. 6, *C* and *D*).

### Statistical procedures

The numbers of biological replicates for each experiment are always indicated in the figure legends. Numerical values of the luminescence data (rlu cm^−1^) of the bacterial growth assays were log_10_-transformed and subject to ANOVA with *post hoc* Tukey’s HSD test (*p* < 0.05 for each data subset; (10)). For metabolite and RT-qPCR-derived gene expression results, non-transformed numerical values were analyzed by ANOVA with *post hoc* Tukey’s HSD test (*p* < 0.05) or by a non-parametric one-way ANOVA according to Kruskal-Wallis with stepwise step-down comparisons (*p* < 0.05). ANOVA analyses were performed with the SPSS statistical software (version 26; IBM Corporation). Additionally, for pairwise comparisons between control- and treatment samples, a two-tailed Student’s *t* test was performed using Microsoft Excel. The depicted results were confirmed in at least one other independent experiment.

## Data availability

All data are contained within the manuscript.

## Supporting information

This article contains supporting information (73, 94, 95, 96, 97).

## Conflict of interest

The authors declare that they have no conflict of interest with the contents of this article.
