# Supplementary material for: In-depth analysis of isochorismate synthase-derived metabolism in plant immunity: Identification of meta-substituted benzoates and salicyloyl-malate
Source: J Biol Chem. 2024 Aug 12;300(9):107667. doi: 10.1016/j.jbc.2024.107667 (PMC11416591; doi:10.1016/j.jbc.2024.107667)
Supplement: Supporting information [file mmc1.pdf]

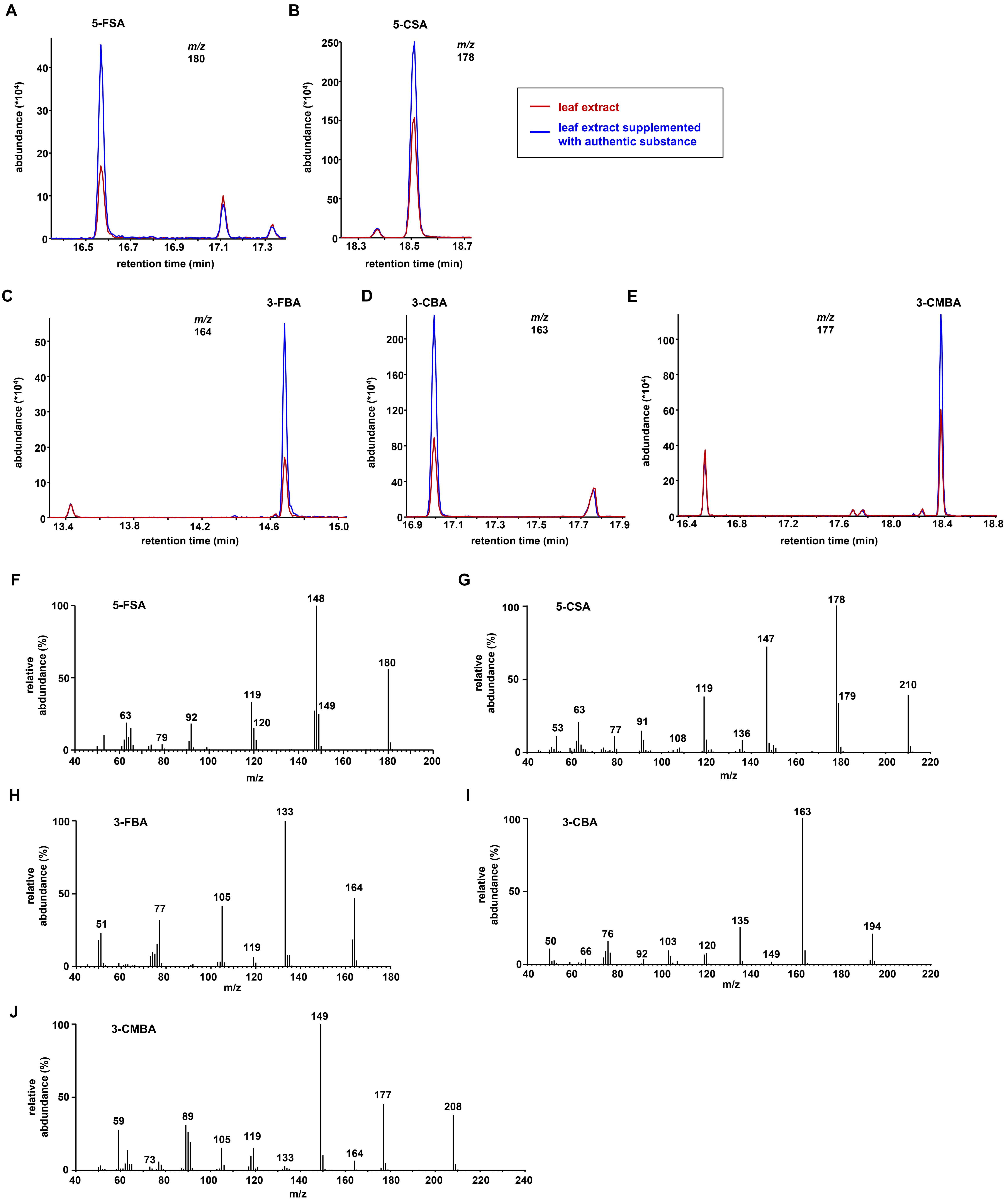

Supporting Figure 1. The *meta*-substituted salicylic acid- and benzoic acid-derivatives detected in plant extracts and authentic compounds possess identical GC retention times and mass spectra. *A-E*, Overlays of ion chromatograms of a GC-MS sample from extracts of *Psm*-inoculated Arabidopsis *npr1* leaves (red) and the same leaf extract sample supplemented with 100-200 ng of the indicated authentic substances. The co-injection of extract sample and authentic compounds yield single peaks with identical retention times. *A*, 5-formyl-salicylic acid (5-FSA, 1). *B*, 5-carboxy-salicylic acid (5-CSA, 2). *C*, 3-formyl-benzoic acid (3-FBA, 4). *D*, 3-carboxy-benzoic acid (3-CBA, 5). *E*, 3-carboxymethyl-benzoic acid (3-CMBA, 6). *F-J*, Mass spectra of authentic, trimethylsilyl-diazomethane-derivatised 5-FSA (*F*), 5-CSA (*G*), 3-FBA (*H*), 3-CBA (*I*), and 3-CMBA (*J*) are identical to the mass spectra of the plant-derived meta-substituted SA/BA-derivates (Fig. 1).

A

compound	abbre-viation	#	RT (min)	sum formular	$[M-H]^-$	fragment ion	remark	authentic compound
salicylic acid	SA	-	7.30	$C_7H_6O_3$	137.0244	93.0346	$[M-H]^-$ - CO_2	yes
5-formyl-SA	5-FSA	1	5.25	$C_8H_6O_4$	165.0193	121.0295	$[M-H]^-$ - CO_2	yes
5-carboxy-SA	5-CSA	2	5.10	$C_8H_6O_5$	181.0142	137.0244	$[M-H]^-$ - CO_2	yes
5-carboxymethyl-SA	5-CMSA	3	5.71	$C_9H_8O_5$	195.0299	151.0401	$[M-H]^-$ - CO_2	no
3-formylbenzoic acid	3-FBA	4	5.86	$C_8H_6O_3$	149.0244	105.0346	$[M-H]^-$ - CO_2	yes
3-carboxy-BA	3-CBA	5	5.38	$C_8H_6O_4$	165.0193	121.0295	$[M-H]^-$ - CO_2	yes
3-carboxymethyl-BA	3-CMBA	6	5.75	$C_9H_8O_4$	179.035	135.0452	$[M-H]^-$ - CO_2	yes
salicyloyl-malate	SA-Mal	7	7.10	$C_{11}H_{10}O_7$	253.0354	137.0244	-	yes
salicyloyl-aspartate	SA-Asp	8	5.74	$C_{11}H_{11}NO_6$	252.0514	135.0452	-	no
SA-β-glucoside	SAG	-	4.01	$C_{13}H_{16}O_8$	299.0772	137.0244	-	yes
SA-glucose ester	SGE	-	5.16	$C_{13}H_{16}O_8$	299.0772	137.0244	-	no

B

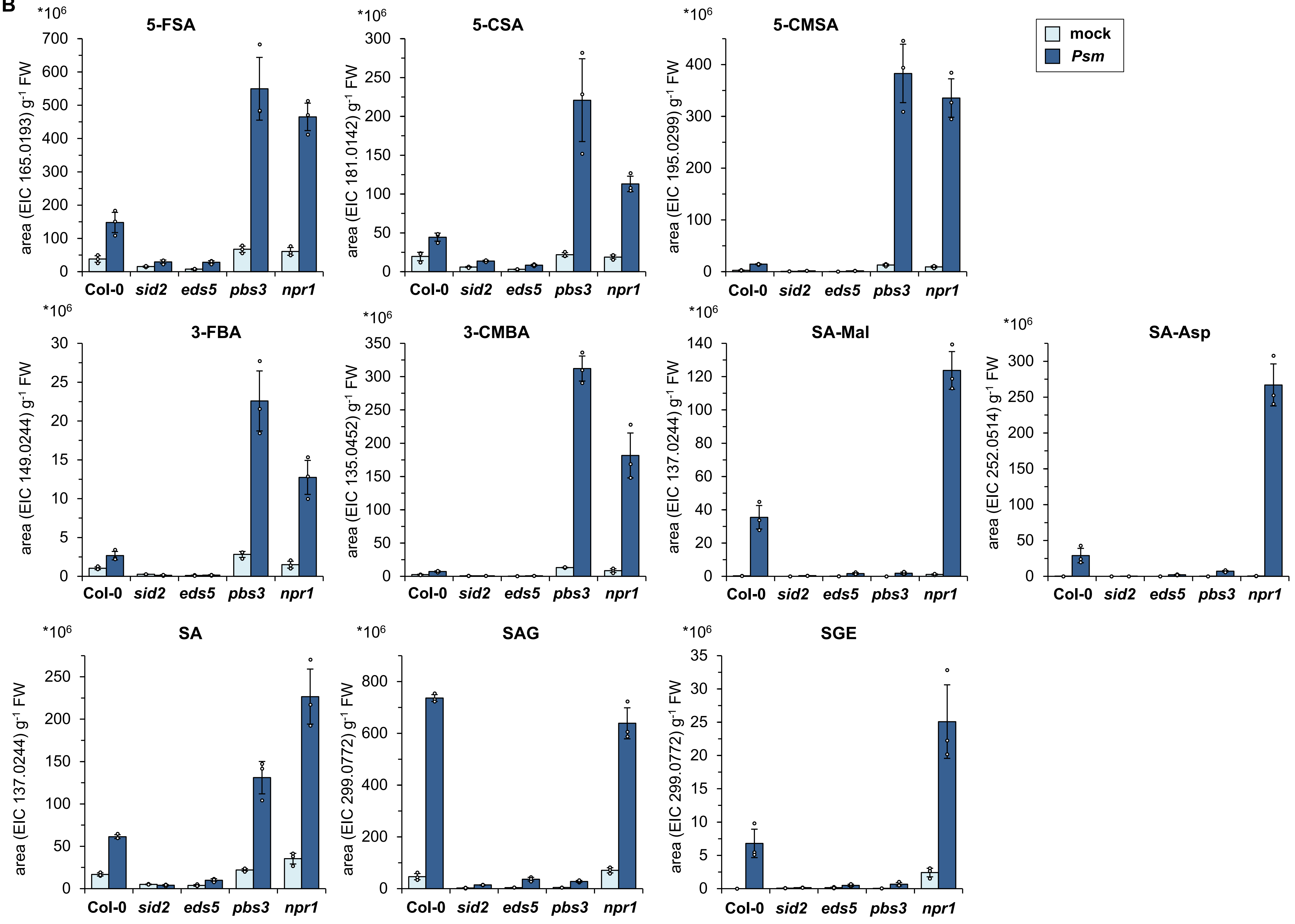

Supporting Figure 2. Analysis of *meta*-substituted SA- and BA-derivatives, SA, and the SA conjugates SA-Mal, SA-Asp, SAG, and SGE by an LC-qTOF-MS-based method. *A*, Table listing retention times (RT), sum formulars, $[M-H]^-$ ions, and other characteristic fragment ions of the compounds analysed in Arabidopsis leaf extracts by LC-qTOF-MS in negative ionization mode (see experimental procedures for details). The last columns indicates whether authentic compounds were available for the confirmation of compound identities. *B*, Relative quantification of the indicated ICS1-derived substances in leaves of Col-0, *sid2*, *eds5*, *pbs3*, and *npr1* plants via LC-qTOF-MS-based analysis of methanolic ($MeOH/H_2O$ = 80:20) leaf extracts. Areas of extracted ion chromatograms (EIC) of the indicated $[M-H]^-$ ions or characteristic fragment ions were determined and related to the fresh weight (FW) of the leaf material. Each sample is derived from the extraction of *Psm*- (dark blue bars) and mock-inoculated (light blue bars) leaves at 48 h post treatment. The bars represent the mean ± SD of three biological replicates from different plants, each replicate consisting of six leaves from two plants. Individual data points of biological replicates are super-imposed on the bar graphs (small circles). Please note the similar accumulation patterns of the analytes in the data sets derived from GC-MS- (Fig. 4) and LC-qTOF-MS-based analysis. Due to the identical masses and similar retention times of 5-FSA and 3-CBA and a consequent overlap of peaks in the EICs, an exact quantification of 3-CBA was not possible via LC-qTOF-MS and therefore not presented in *B*.

Supporting Figure 3. Chemical synthesis and spectroscopic characterization of salicyloyl-malate (SA-Mal)

Synthesis of *rac-O*-salicyloyl malic acid (6) from *rac*-malic acid (1).

The synthesis of the *rac*-*O*-salicyloyl malic acid (**6**) commences with the acid catalyzed benzylation of *rac*-malic acid (**1**) with benzyl alcohol (**2**) to give *rac*-dibenzyl malate (**3**) in 71% yield as a pale-yellow oil according to literature (Scheme 1).[1] Dibenzyl malate (**3**) undergoes Steglich esterification with *O*-benzyl salicylic acid (**4**), which obtained by a literature protocol in two steps from methyl salicylate by benzylation with benzyl bromide and subsequent alkaline saponification,[2] to furnish in analogy to a literature protocol[3] the tribenzylated product **5** in excellent yield. Finally, the target molecule **6** is obtained by global hydrogenolytic debenzylation[4] and crystallization as a colorless solid in 95% yield.

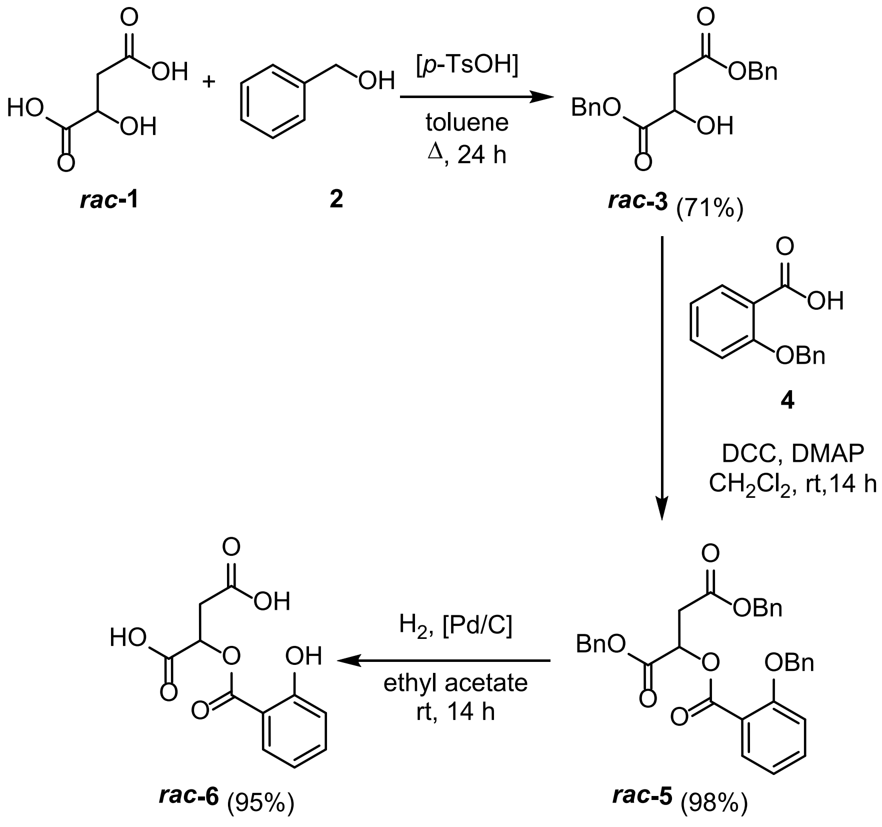

Scheme 1. Synthesis of *rac*-*O*-salicyloyl malic acid (**6**) from *rac*-malic acid (**1**).

Experimental

Synthesis of dibenzyl *rac*-2-hydroxysuccinate (*rac*-3)[1]

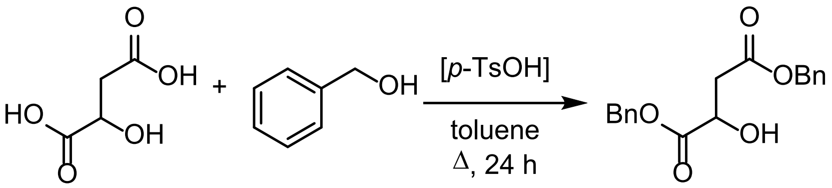

***rac*-1** **2** ***rac*-3** (71%)

***rac*-3** ($C_{18}H_{18}O_5$ [314.33])

Racemic malic acid (**1**) (3.35 g, 25.0 mmol, 1.00 equiv) was placed under nitrogen atmosphere in a screw cap Schlenk tube with magnetic stir bar and dissolved in toluene (40 mL). To the solution was added benzyl alcohol (**2**) (5.41 g, 50.0 mmol, 2.00 equiv), *p*-toluene sulfonic acid (0.047 g, 0.025 mmol, 1.00 mol%) and the mixture was heated heated to reflux for 24 h. After cooling to room temp, the solvent was removed under reduced pressure and the crude product was purified by column chromatography on silica gel (n-hexane/ethyl acetate 5:1) to give dibenzyl *rac*-2-hydroxysuccinate (***rac*-3**) (6.01 g, 19.1 mmol, 71%) as a pale-yellow oil, R_f (n-hexane/ethyl acetate 5:1): 0.13.

Synthesis of *O*-benzyl salicylic acid (4)[2]

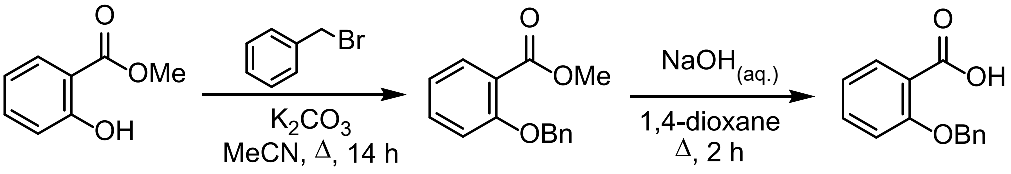

4 (80%)

4 ($C_{14}H_{12}O_3$ [228.24])

Methyl 2-hydroxybenzoate (28.0 g, 180 mmol, 1.00 equiv) was placed in a 1000 mL round bottom flask with magnetic stir bar and a reflux condenser and dissolved in acetonitrile (750 mL). Potassium carbonate (49.5 g, 360 mmol, 2.00 equiv) was added under vigorous stirring and the reaction mixture was stirred at room temp for 10 min. Benzyl bromide (32.4, 193 mmol, 1.07 equiv) was then added to the mixture with constant stirring and the reaction mixture was heated to reflux for 14 h. After cooling to room temp, the solvent was removed under reduced pressure. The crude product was dissolved in water (150 mL) and extracted with ethyl acetate (3 × 25 mL). The collected organic layers were dried with anhydrous sodium sulfate, filtered and then the solvents were removed under reduced pressure. The crude product was dissolved in 1,4-dioxane (50 mL) and aqueous sodium hydroxide solution (1.5 M, 60.0 mL) was added. The mixture was heated to reflux for 2 h and then the solvent was removed under

reduced pressure. The aqueous phase was then extracted with ethyl acetate (3x 50 mL). The organic phase was dried with anhydrous sodium sulfate, then filtered and the solvent removed under reduced pressure to give *O*-benzyl salicylic acid (**4**) (32.5 g, 143 mmol, 80%) as a yellowish solid, R_f (n-hexane/ethyl acetate 3:1) = 0.90.

MS-ESI *m/z* (100%): 229 ([M+H]$^+$, 100), 457 ([2M+H]$^+$, 15), 495 ([2M+K]$^+$, 10).

Synthesis of dibenzyl *rac*-2-{[2-(benzyloxy)benzoyl]oxy}succinate (*rac*-5) (according to ref. 3)

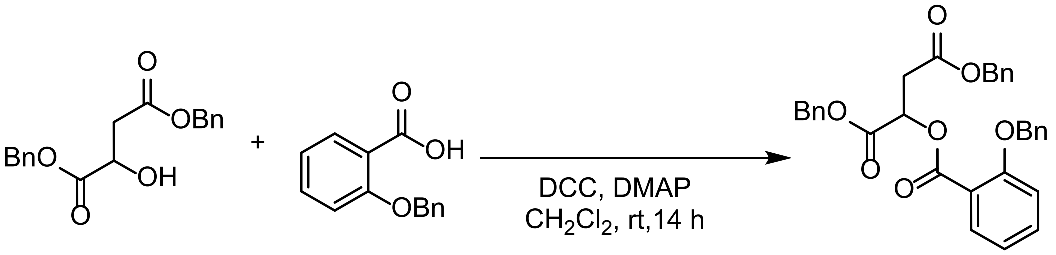

***rac*-3** (71%) **4** ***rac*-5** (98%)

***rac*-5** ($C_{32}H_{28}O_7$ [524.57])

Dibenzyl *rac*-2-hydroxysuccinate (***rac*-3**) (1.16 g, 5.04 mmol, 1.00 equiv) was placed in a 50 mL round bottom flask with magnetic stir bar and dissolved in dichloromethane (25 mL). Subsequently, *O*-benzyl salicylic acid (**4**) (1.74 g, 5.55 mmol, 1.10 equiv), dicyclohexylcarbodiimide (DCC) (1.14 g, 5.55 mmol, 1.10 equiv), and 4-(dimethylamino)pyridine (DMAP) (0.065 g, 0.504 mmol, 0.10 equiv) were added. A suspension was formed immediately. The reaction mixture was stirred at room temp for 14 h. The solvent was removed under reduced pressure and the crude product was then purified by column chromatography (*n*-hexane/ethyl acetate 3:1) to give dibenzyl *rac*-2-{[2-(benzyloxy)benzoyl]oxy}succinate (***rac*-5**) (2.59 g, 4.93 mmol, 98%) as a colorless resin, R_f (*n*-hexane/ethyl acetate 3:1) = 0.41.

MS ESI: *m/z* (%): 525 ([M + H]$^+$, 100), 563 ([M + K]$^+$, 10).

Synthesis of *rac-O*-salicyloyl malic acid (*rac*-6) (according to ref. 4)

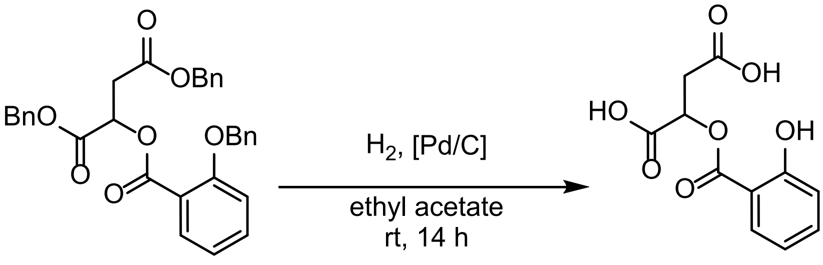

***rac*-5** (98%) ***rac*-6** (95%)

***rac*-6** ($C_{11}H_{10}O_7$ [254.19])

In a Schlenk tube with a rubber septum and magnetic stir bar, *rac*-2-{[2-(benzyloxy)benzoyl]oxy}succinate (***rac*-5**) (2.00 g, 3.81 mmol, 1.00 equiv) were placed 10 wt% Pd/C (40 mg, 40 mmol) and ethyl acetate (20 mL). The reaction mixture was purged with a stream of nitrogen for 1 min. Via a cannula, hydrogen gas was passed through the septum into the reaction mixture (approx. 180 mL of gas until the solution was saturated). The reaction mixture was stirred at room temp for 14 h. The reaction mixture was filtered, the solvent was removed under reduced pressure, and the crude solid product was crystallized from dichloromethane/acetone (50:1) to give *rac-O*-salicyloyl malic acid (***rac*-6**) (0.92 g, 3.61 mmol, 95%) as a colorless solid, R_f (*n*-hexane/ethyl acetate 3:1) = 0.41.

^{1}H NMR (600 MHz, acetone-d$_6$): δ 3.09-3.20 (m, 2H), 5.77 (dd, J = 7.6, 4.5 Hz, 1H), 6.88-7.06 (m, 2H), 7.57 (ddd, J = 8.7, 7.2, 1.8 Hz, 1H), 7.93 (dd, *J* = 8.0, 1.8 Hz, 1H), 10.40 (s, 1H), 11.50 (s, 2H). ^{13}C NMR (150 MHz, acetone-d$_6$): δ 36.2 (CH_2), 70.1 (CH), 112.8 (C_{quat}), 118.4 (CH), 120.3 (CH), 131.1 (CH), 137.2 (CH), 162.5 (C_{quat}), 169.7 (C_{quat}), 169.8 (C_{quat}), 179.8 (C_{quat}). MS ESI *m*/*z* (%): 547 ([2M + K]$^+$, 100). Anal. calcd. for [$C_{11}H_{10}O_7$]: C 51.98, H 3.97; Found: C 51.32, H 3.94.

NMR Spectra of *rac*-*O*-salicyloyl malic acid (*rac*-6)

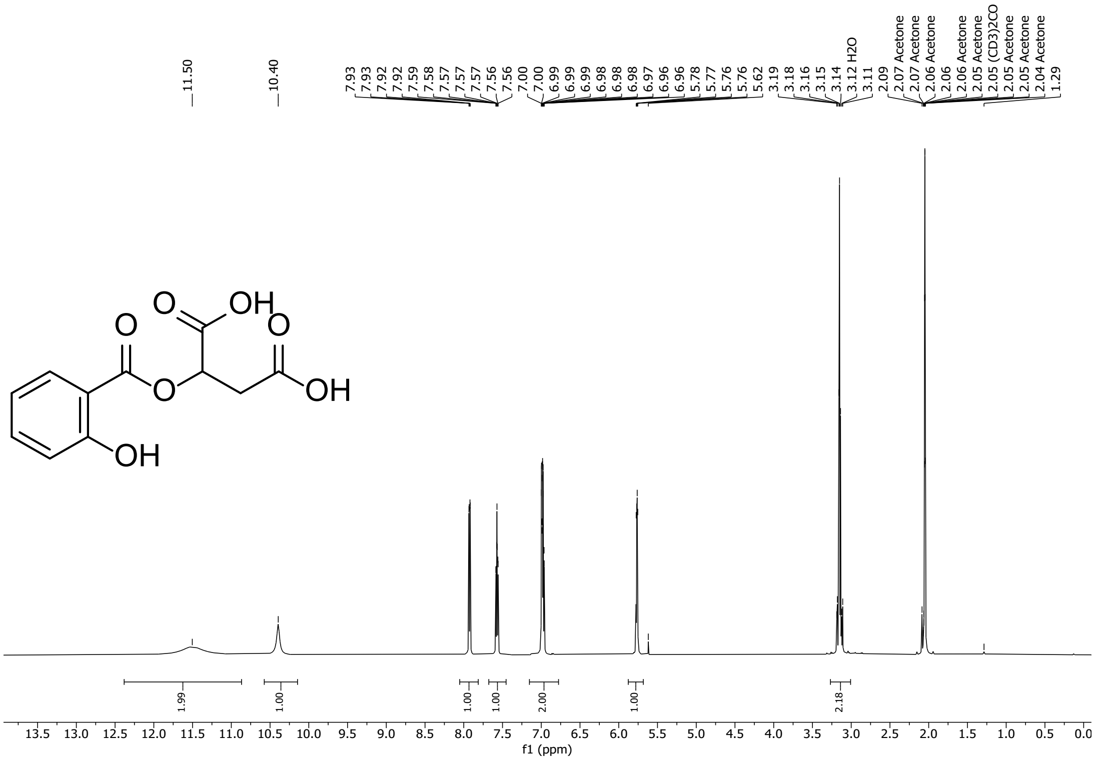

A) ^{1}H NMR (600 MHz, acetone-d_6) spectrum of *rac*-*O*-salicyloyl malic acid (***rac*-6**).

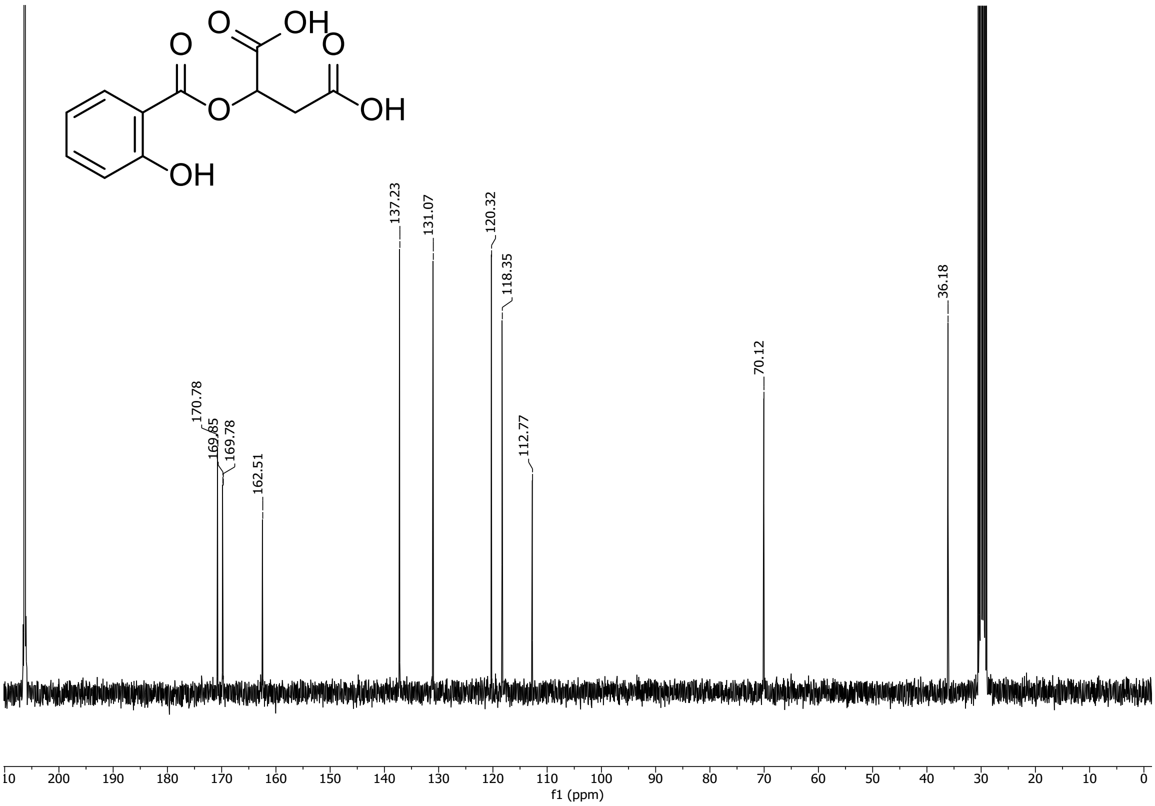

B) ^{13}C NMR (150 MHz, acetone-d_6) spectrum of of *rac*-*O*-salicyloyl malic acid (***rac*-6**).

Fig. S3

References

[1] S. S. Lee, Z.-H. Li, D. H. Lee, D. H. Kim, (2R,3S)- and (2S,3R)-2-Benzyl-3,4-epoxybutanoic acid as highly efficient and fast acting pseudomechanism-based inactivators for carboxypeptidase A: design, asymmetric synthesis and inhibitory kinetics. *J. Chem. Soc., Perkin Trans. 1* **1995**, 2877-2882. https://doi.org/10.1039/P19950002877

[2] S.-T. Huang, I. Hsei, C. Chen, Synthesis and anticancer evaluation of bis(benzimidazoles), bis(benzoxazoles), and benzothiazoles. Bioorg. Med. Chem. **2006**, *14*, 6106-6119. https://doi.org/10.1016/j.bmc.2006.05.007

[3] C. M. Krell, D. Seebach, Preparation of Free and of Specifically Protected Oligo[β-Malic Acids] forEnzymatic Degradation Studies. *Eur. J. Org. Chem.* **2000**, 1207-1218. https://doi.org/10.1002/1099-0690(200004)2000:7<1207::AID-EJOC1207>3.0.CO;2-2

[4] T. Fujisawa, M. Kurosawa, T. Katagi, Uptake and Transformation of Pesticide Metabolites by Duckweed (*Lemna gibba*), J. Agric. Food Chem. **2006**, *54*, 6286-6293. https://doi.org/10.1021/jf061301g

Fig. S3

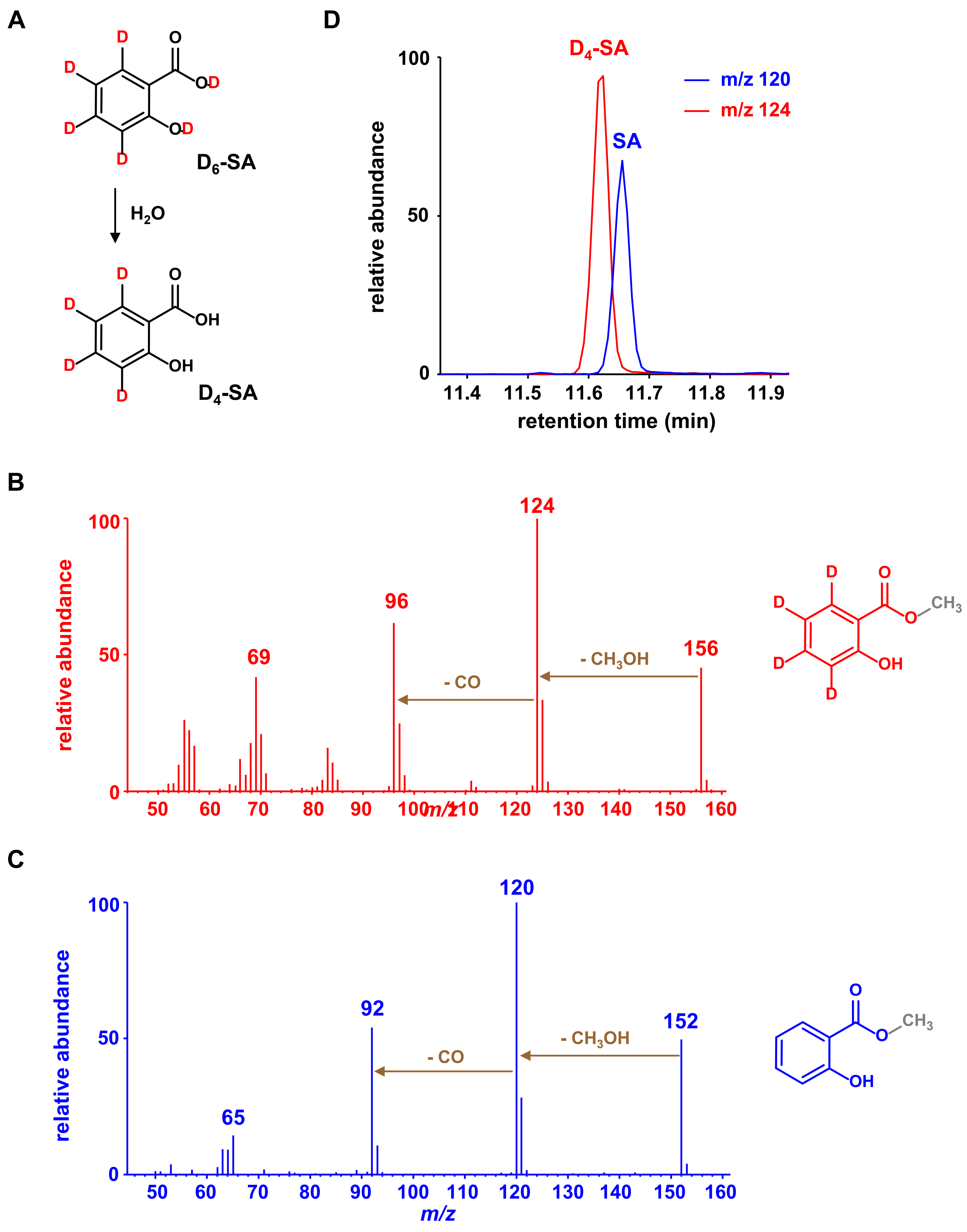

Supporting Figure 4. Hydrogen-deuterium exchange at labile carboxylic and phenolic positions converts the applied deuterated D$_6$-SA into D$_4$-SA in aqueous solvents. *A*, The six-fold deuterated salicylic acid (D$_6$-SA) applied for labelling experiments is rapidly converted to D$_4$-SA in aqueous and other protic solvents by hydrogen-deuterium exchange. Thus, D$_4$-SA is the actually applied form of deuterated SA. *B*, Mass spectrum and fragmentation pattern of D$_4$-SA generated by dissolving D$_6$-SA in aqueous solvents for the preparation of stock and working solutions. *C*, Mass spectrum and fragmentation pattern of unlabeled SA for comparison. *D*, Ion chromatograms of *m/z* 124 and *m/z* 120 in GC-MS sample from Arabidopsis leaf extracts, indicating the presence of exogenously added D$_4$-SA and endogenously accumulating SA in the extract samples. Plant metabolic activities further converted (D$_4$)-SA into (D$_4$)-SA conjugates such as (D$_4$)-SA-Mal or (D$_4$)-SA-Asp (Figs. 2 and 3). Note that the samples were derivatised with trimethylsilyl-diazomethane to convert carboxylic acid groups into methyl ester groups prior to GC-MS analysis. The methyl group introduced by derivatisation is indicated in grey.

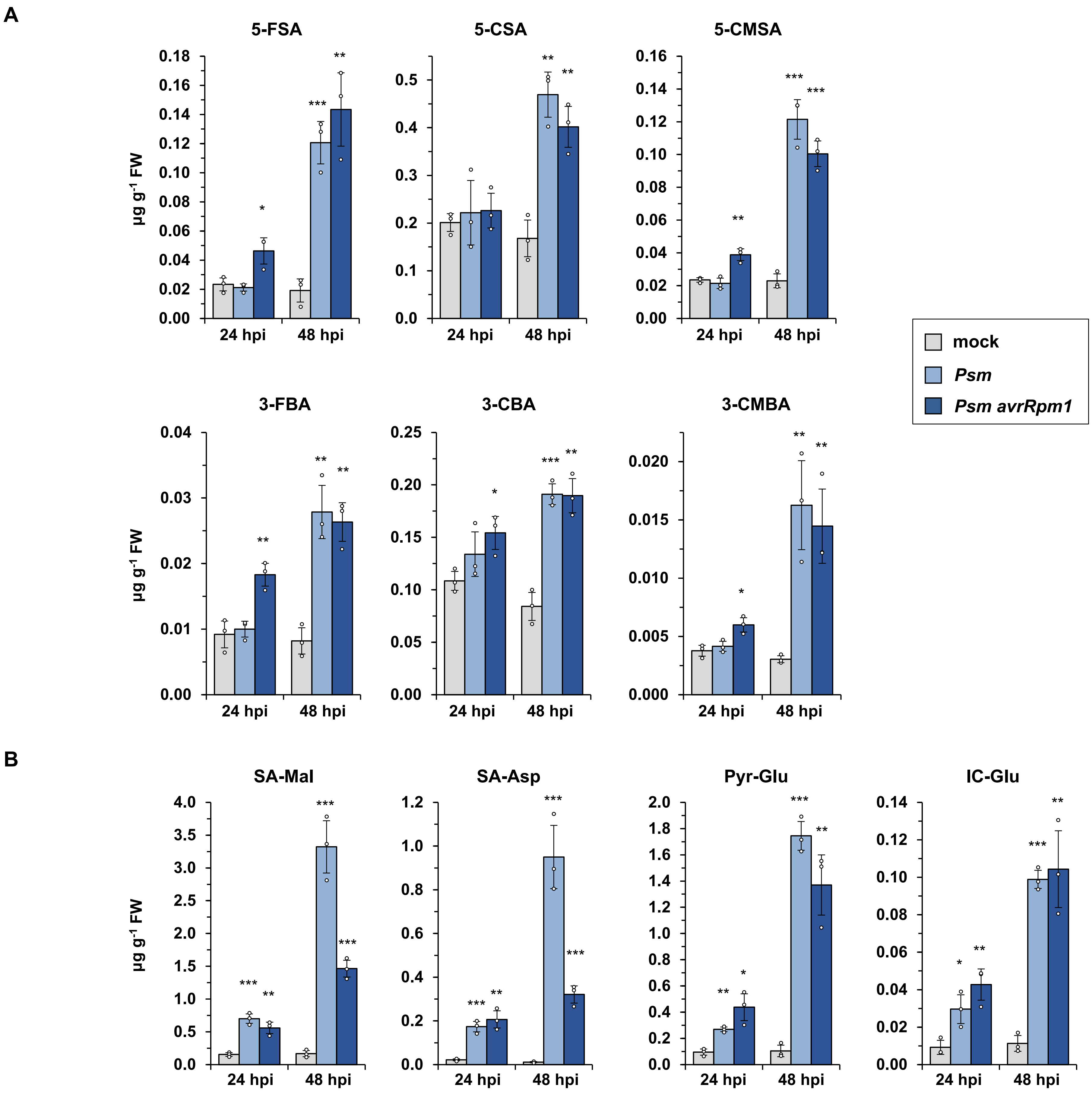

Supporting Figure 5. *meta*-Substituted SA/BA-derivatives and other SA-related metabolites accumulate in both compatible and incompatible plant-bacterial interactions. Levels of different *ICS1*-dependent metabolites at 24 and 48 hours post inoculation (hpi) with a suspension of OD_{600} = 0.005 of the compatible (virulent) *Psm* strain (*Psm*), a suspension of OD_{600} = 0.005 of the incompatible (avirulent) *Psm avrRpm1* strain (*Psm avrRpm1*), or mock-inoculation (mock) by infiltrating an aqueous, 10 mM $MgCl_2$ solution. Three rosette leaves per plant were infiltrated, and metabolite contents determined by GC-MS analysis as described in the text. Metabolite levels are given in µg per gram FW. Bars represent means ± SD of three biological replicates. One replicate sample consisted of six leaves from two plants. Individual data points of biological replicates are super-imposed on the bar graphs (small circles). The presence of asterisks above the lines indicates whether significant differences between mock- and bacterial inoculations exist (***P < 0.001, **P < 0.01, *P < 0.05; two-tailed *t*-test). Absence of asterisks indicates no significant difference (P > 0.05). *A*, *Meta*-substituted SA/BA-derivatives: 5-FSA, 5-CSA, 5-CMSA, 3-FSA, 3-BSA, and 3-CMBA. *B*, SA-Mal, SA-Asp, Pyr-Glu, and IC-Glu. Related to Fig. 5.

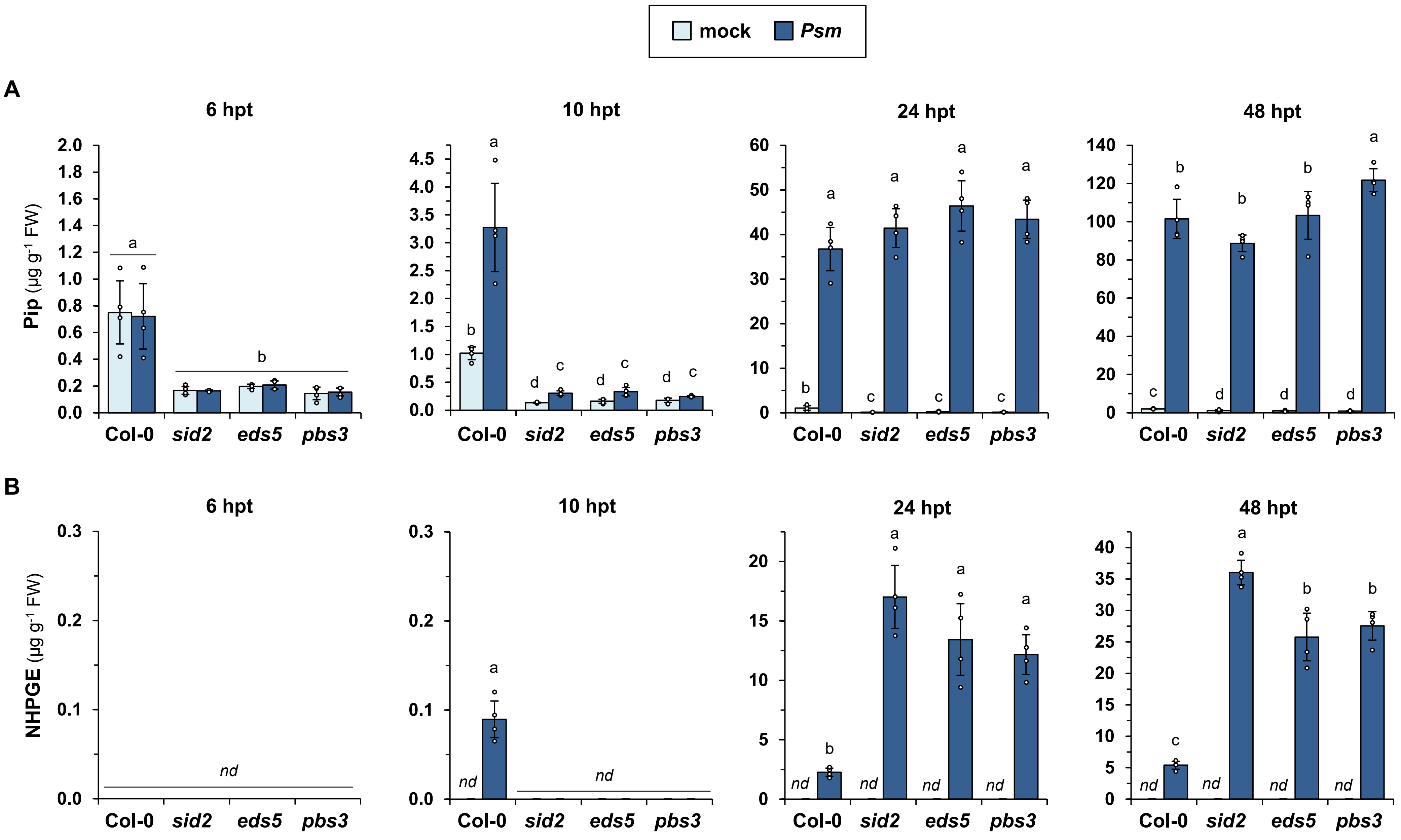

Supporting Figure 6. Time course of accumulation of pipecolic acid and NHP glucose ester in *P. syringae*-inoculated Col-0, *sid2*, *eds5*, and *pbs3* plants. *A-B*, Levels of (*A*) pipecolic acid (Pip) and (*B*) *N*-hydroxypipecolic acid glucose ester (NHPGE) at 6, 10, 24, and 48 hours post treatment (hpt) in mock-infiltrated (mock) and *Psm*-inoculated (*Psm*) leaves of Arabidopsis Col-0, *sid2*, *eds5*, and *pbs3* plants. Three rosette leaves per plant were treated. Metabolite contents were determined by GC-MS analysis of trimethyl-silylated analytes and are given in µg per gram FW. Bars represent means ± SD of four biological replicates. One replicate sample consisted of six leaves from two plants. Individual data points of biological replicates are super-imposed on the bar graphs (small circles). Different letters denote significant differences ($p < 0.05$, Kruskal-Wallis H test). *nd*: not detected. Related to Fig. 7.

Gene		Primer sequence (5' to 3')	
At2g14610	*PR1*	Forward primer<br>Reverse primer	GTGCTCTTGTTCTTCCCTCG<br>GCCTGGTTGTGAACCCTTAG
At1g43190	*PTB* (reference)	Forward primer<br>Reverse primer	GATCTGAATGTTAAGGCTTTTAGCG<br>GGCTTAGATCAGGAAGTGTATAGTCTCTG

Supporting Figure 7. Primers used for RT-qPCR-analyses.

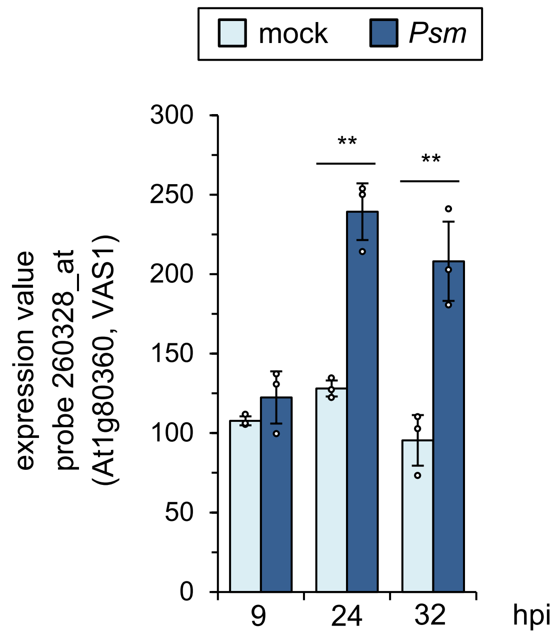

Supporting Figure 8. Expression levels of *VAS1* in Arabidopsis leaves inoculated with *Psm* according to publicly available microarray analyses. Means ± SD of Affymetrix expression values of probe 260328_at (At1g80360, *VAS1*) originating from three biological replicates are given. Individual expression values of biological replicates are super-imposed on the bar graphs (small circles). The presence of asterisks above the lines indicates whether significant differences between mock- and *Psm*-treatment exist for the particular timepoint (***P < 0.001, **P < 0.01, *P < 0.05; two-tailed *t*-test). The data originate from NASCARRAYS-414: Gene expression in Arabidopsis leaves inoculated with *Pseudomonas syringae* pv. *maculicola* ES4326. Individual leaves of Col-0 plants were infiltrated with *Psm* (OD_{600} = 0.002) or mock-infiltrated and harvested 9, 24 or 32 hpi (Nafisi et al., 2007).
